# Unraveling the reasons behind lead phthalocyanine acting as a good absorber for near-infrared sensitive devices

**DOI:** 10.1038/s41598-022-12990-z

**Published:** 2022-05-25

**Authors:** Masahiro Kato, Hayato Yoshizawa, Masato Nakaya, Yasutaka Kitagawa, Koichi Okamoto, Tomoaki Yamada, Masahito Yoshino, Kentaro Tanaka, Jun Onoe

**Affiliations:** 1 Department of Energy Science and Engineering, Nagoya University, Furo-cho, Chikusa-ku, Nagoya, 464-8603 Japan; 2 Graduate School of Engineering Science, Osaka University, Machikaneyama, Toyonaka, Osaka 560-8531 Japan; 3 Department of Physics and Electronics, Osaka Prefecture University, Gakuen-cho, Naka-ku, Sakai, Osaka 599-8531 Japan; 4 Department of Chemistry, Nagoya University, Furo-cho, Chikusa-ku, Nagoya, 464-8602 Japan

**Keywords:** Solar cells, Energy

## Abstract

Lead phthalocyanine (PbPc) is well known to be used as a good near-infrared (NIR) light absorber for organic solar cells (OSCs) and photodetectors. The monoclinic and triclinic phases have been understood to absorb the visible and NIR regions, respectively, so far. In the present study, we demonstrated from the absorption spectra and theoretical analysis that the visible band considerably originates from not only the monoclinic but also the amorphous and triclinic phases, and revealed the exciton dynamics in the PbPc film from static/time-resolved photoluminescence (PL), which are first reported. By comparing the external quantum efficiency between PbPc- and ZnPc-based OSCs in relation to their structure, morphology, and optical (absorption and PL) characteristics, we unraveled the reasons behind the PbPc film used as a good absorber for NIR-sensitive devices.

## Introduction

**Figure 1:**
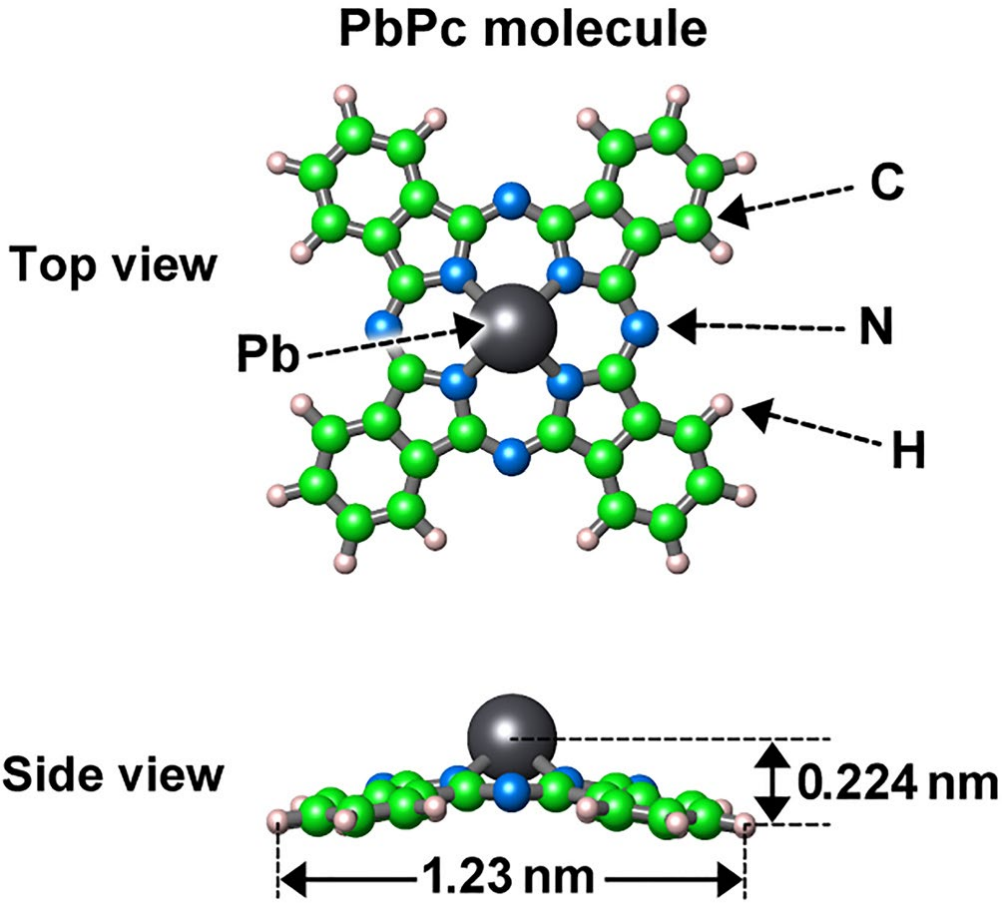
Schematic illustration of the top and side views of the PbPc molecule.

**Figure 2:**
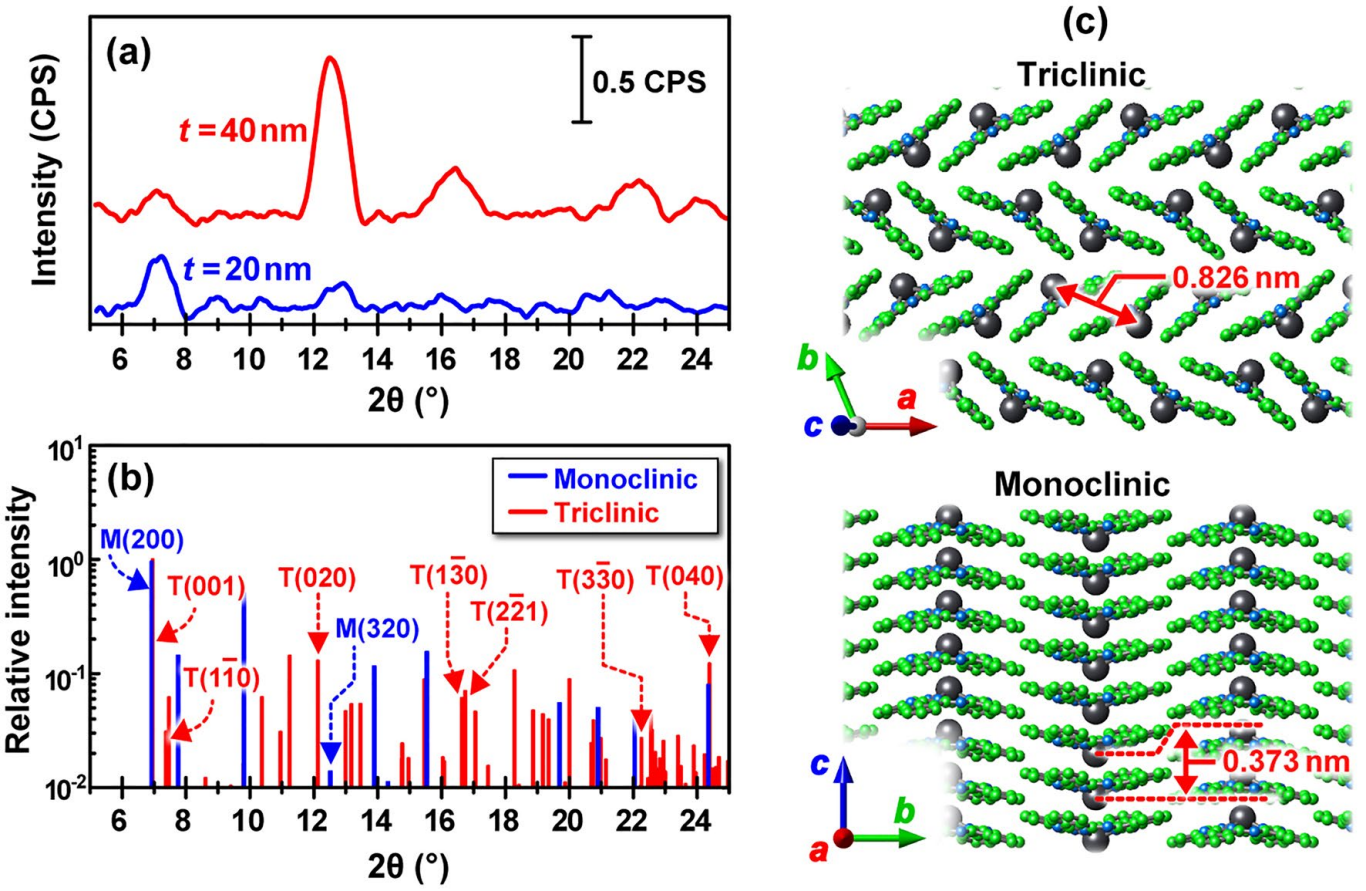
(**a**) XRD patterns of the 20 nm (blue) and 40 nm (red) thick PbPc films formed on quartz substrates, (**b**) simulated XRD patterns of the monoclinic (blue) and triclinic (red) crystal phases using an X-ray wavelength of 1.5405 nm (Cu-Kα1) [the maximum peak intensity of both phases was individually normalized to unity], (**c**) schematic illustration of the triclinic (top) and monoclinic (bottom) crystal phases.

Lead phthalocyanine (PbPc) has been extensively used as a good near-infrared (NIR) light absorber for NIR-sensitive organic solar cells (OSCs)^1–11^. As shown in Fig. 1, the PbPc molecule is well known to have a nonplanar unique structure called a “shuttle cock”. This shuttle cock shape leads to the formation of the monoclinic and triclinic crystal phases of PbPc films (see Fig. 2c).

In particular, the latter phase has been understood to play a key role in absorbing the NIR light region^5,12^. However, recent theoretical analysis^13^ suggests that the NIR absorption band of the PbPc film after annealing is attributed to the monoclinic phase rather than the triclinic phase, although the annealed film is well known to have the triclinic phase experimentally^3,14^. In addition, to the best of our knowledge, although there have been many reports to discuss the performance of PbPc-based OSCs in relation to the absorption spectra of those phases so far^1–11^, there are no reports to discuss the performance of PbPc-based OSCs in relation to the photoluminescence (PL) spectra of the monoclinic and triclinic phases, which provide useful information on the exciton characteristics generated in PbPc films. This may be because the PL intensity of the PbPc film is too weak to be detected using conventional PL measurement systems with a monochromatized UV–vis lamp. Furthermore, although the exciton diffusion length (*L*_D_ = 4.6 nm) of the PbPc films^15^ is much shorter than that (*L*_D_ = 15 nm) of ZnPc films^16^, the external quantum efficiency (*EQE*) of the PbPc-based OSC is almost twice that of the ZnPc-based OSC (see Fig. 6) in the visible region at approximately 700 nm, although their absorbances in the region are comparable to each other (see Fig. S6). To reveal the reasons behind these conflicting results, it is necessary to examine the structural, morphological, and optical (not only absorption but also PL) characteristics of PbPc films more precisely.

In the present study, we investigated the structural, morphological, and optical characteristics of PbPc films using X-ray diffraction (XRD), atomic force microscopy (AFM), UV–vis-NIR absorption spectroscopy, and laser-induced static/time-resolved (TR) PL spectroscopy, in combination with first-principles calculations based on density functional theory, and discussed the difference in the *EQE* between [ITO/PbPc/C_60_/Al] and [ITO/ZnPc/C_60_/Al] OSCs to unravel the reasons behind the conflicts on the assignment of the absorption bands of the monoclinic and triclinic phases between the experimental and theoretical results and behind PbPc acting as a good absorber for NIR-sensitive OSCs.

## Results and discussion

### X-ray diffraction (XRD)

**Figure 3:**
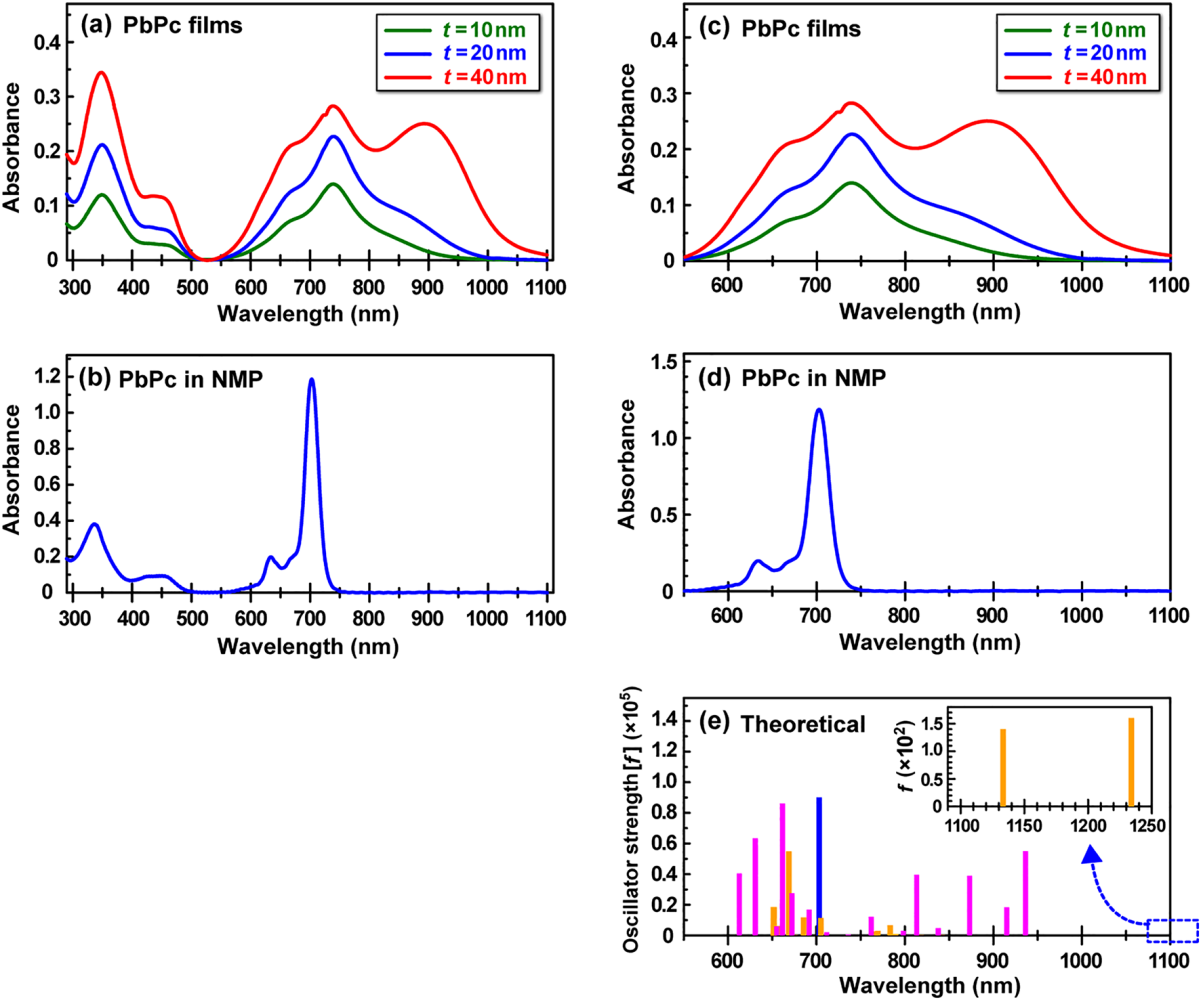
(**a**) UV–vis-NIR spectra of the 10-nm (green), 20-nm (blue), and 40-nm (red) thick PbPc films formed on quartz substrates, (**b**) UV–vis-NIR spectra of PbPc solution (8.5 × 10^−5^ M in NMP solvent) filled in a quartz cell, (**c,d**) the magnified Q-band spectra of (a) and (b), and (**e**) theoretical absorption stick spectra of PbPc isolated molecule (blue) and the monoclinic (yellow)/triclinic (pink) tetramer models (see Fig. 4). Here, the theoretical stick spectra of the isolated PbPc molecule were obtained by considering the solvent effects (the relative permittivity of NMP is almost equal to that of methanol).

Figure 2 shows (a) XRD patterns of the 20 nm (blue) and 40 nm (red)-thick PbPc films formed on quartz substrates at room temperature (RT) and at a deposition rate of 0.42 nm/min (0.07 Å/s), (b) simulated XRD patterns of the monoclinic (blue) and triclinic (red) crystal phases, and (c) schematic illustration of the triclinic (top) and monoclinic (bottom) crystal phases. A comparison between the experimental and simulated XRD patterns suggests that the monoclinic phase was dominant for the 20 nm-thick PbPc film because the peaks corresponding to the triclinic phase were not observed in the range of 2*q* = 16–18°. As the film thickness increased to 40 nm, the peaks attributed to the triclinic phase became appeared remarkably at 2*q* = 12°, 16–18°, and 22–24°. This indicates that the 40 nm-thick PbPc film has a mixture of both phases. Correspondingly, the NIR absorption band (800–1000 nm) increased with the film thickness, as discussed in the next section (see Fig. 3a,c). This is consistent with a previous report that mixed monoclinic-triclinic layers with enhanced NIR absorption were obtained by increasing the thickness^3^.

### UV–vis-NIR absorption spectra

**Figure 4:**
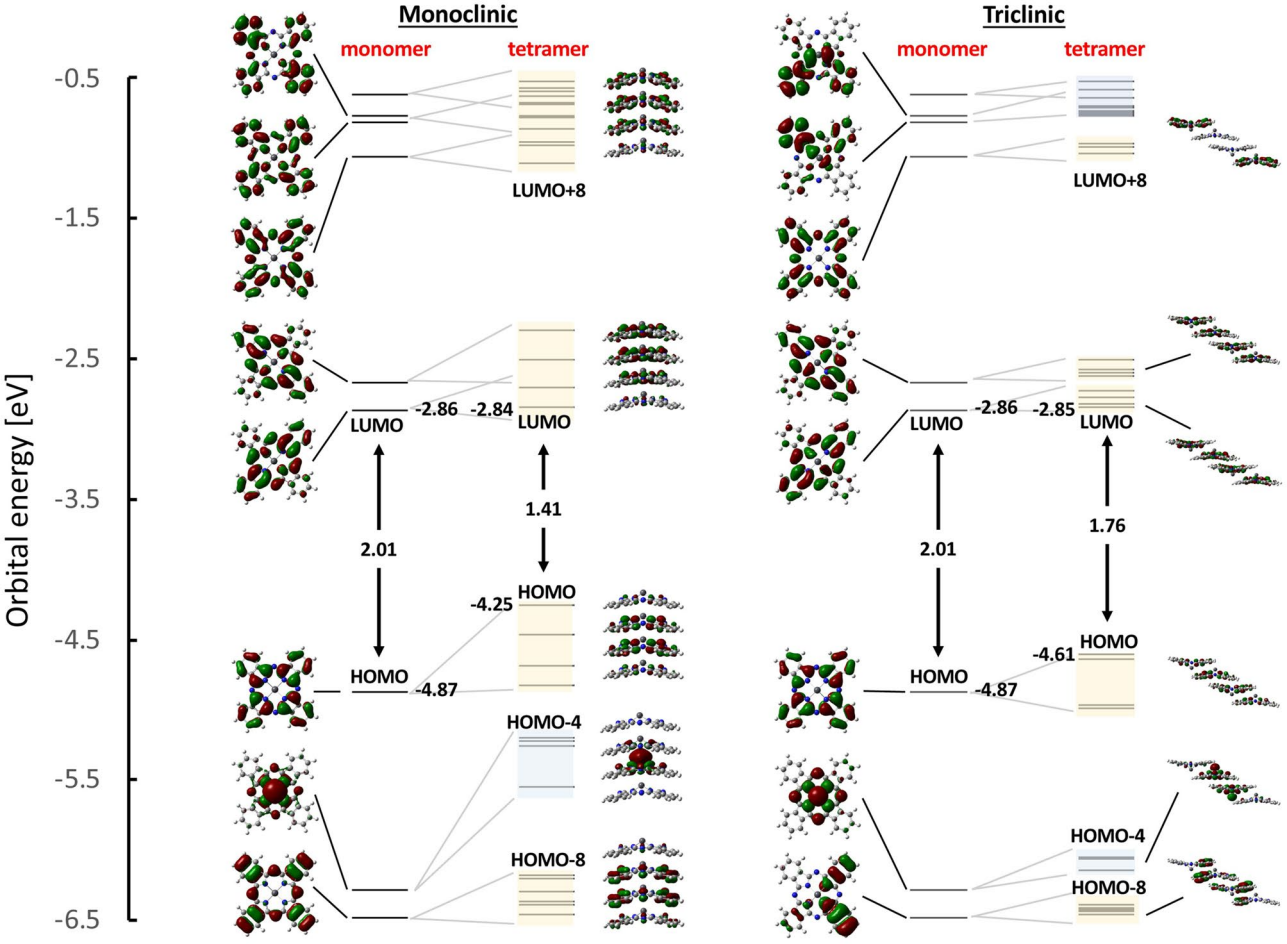
Energy diagram and wavefunctions of the corresponding molecular orbitals (MOs) for the monoclinic and triclinic tetramer models. Here, the bands indicated by light yellow and light blue colours denote p- and d-orbital-based bands, respectively. The HOMO and LUMO are abbreviated expressions of the highest occupied and the lowest unoccupied MOs, respectively.

We next examined the UV–vis-NIR absorption spectra of PbPc films with thicknesses of 10, 20, and 40 nm, along with isolated PbPc molecules dissolved in *N*-methylpyrrolidone (NMP), and compared these spectra with theoretical spectra obtained using first-principles calculations. Figure 3 shows (a) UV–vis-NIR spectra of the 10 nm (green)-, 20 nm (blue)-, and 40 nm (red)-thick PbPc films, (b) UV–vis-NIR spectra of isolated PbPc molecules in NMP (8.5 × 10^−5^ M), (c, d) the magnified Q-band spectra of Fig. 3a and b, respectively, and (e) theoretical absorption stick spectra of PbPc isolated molecule (blue) in NMP and of the monoclinic (yellow)/triclinic (pink) tetramer models (see Fig. 4).

As shown in Fig. 3a, the absorption band in the range of 300–500 nm is assigned to the Soret band, whereas the broad absorption band in the range of 550–1100 nm is assigned to the Q-band. The origins of these bands have often been understood in terms of the four frontier orbital (HOMO–1, HOMO, LUMO, LUMO + 1) model proposed by Gouterman and his coworkers^17–19^. Here, the HOMO and LUMO are the abbreviated expressions of the highest occupied and the lowest unoccupied molecular orbitals, respectively. Since Table S1 (Supplementary) shows that the Q-band excitation wavelength (HOMO → LUMO/LUMO + 1) for the PbPc molecule in solvent (methanol) was redshifted by ca. 20 nm when compared to that for the molecule in the gas phase, a comparison between the experimental (c, d) and theoretical (e) spectra in the Q-band suggests that the intense peak at approximately 700 nm for the monomer (d) corresponds to the most intense peak of the Q-band at approximately 740 nm for the films (c). In a similar manner, a comparison between Fig. 3a and b spectra indicates that the peak at approximately 340 nm for the isolated PbPc monomer (b) corresponds to the most intense peak of the Soret band at approximately 350 nm for the PbPc films (a). These peak shifts are due to the dielectric constant of surrounding molecules (solvent effects)^20^. Thus, these results imply that an amorphous phase, which resembles the state of isolated PbPc molecules, exists in each PbPc film (an amorphous phase cannot appear in the XRD pattern shown in Fig. 2). Accordingly, it is found from the results of Figs. 2 and 3 that the 10 nm- and 20 nm-thick PbPc films consist of amorphous and monoclinic phases, whereas the 40 nm-thick film consists of amorphous, monoclinic, and triclinic phases (the upper 20 nm-thick layer dominantly consists of the amorphous/triclinic phases, whereas the lower 20 nm-thick layer dominantly consists of the amorphous/monoclinic phases).

We next discuss the changes in the Q-band spectral shape with respect to the film thickness (Fig. 3c) by comparison with theoretical results to solve the conflict on the assignment of the visible and NIR absorption bands between the experimental^5,12^ and theoretical^13^ results reported previously. Figure 4 shows the energy diagram of individual molecular orbitals (MOs) and their corresponding typical wavefunctions for the PbPc monoclinic (left) and triclinic (right) tetramers, along with those for the monomer (all MO wavefunctions are shown in Figs. S1–S3). Here, the bands indicated by light yellow and light blue colours denote the p- and d-orbital-based bands, respectively. As shown in Fig. 4, the HOMO–LUMO gap (1.41 eV) of the monoclinic tetramer becomes much narrower than that (1.76 eV) of the triclinic tetramer when compared to that (2.01 eV) of the monomer. Since the monoclinic phase has a more packed structure than the triclinic phase, the p-bands (and d-band) of the monoclinic tetramer become wider than those of the triclinic tetramer, thus reducing the HOMO (p-band)–LUMO (p-band) gap more significantly than for the triclinic phase. Actually, the electronic states of the monoclinic tetramer become more delocalized than those of the triclinic tetramer, as illustrated in Fig. 4. Thus, the monoclinic phase seems to act as an NIR absorber better than the triclinic phase based on their HOMO–LUMO gaps, which is supported by the theoretical analysis reported previously^13^. To solve the apparent conflict between the experimental (Figs. 2 and 3) and theoretical (Fig. 4) results, the intensity of individual excitation states should be examined more precisely for both phases.

Tables S1–S3 (supplementary) summarize the wavelength and oscillator strength (*f*) of the excitation transitions from S0 to S1–S40 and to S1–S30 for the monomer, monoclinic tetramer, and triclinic tetramer, respectively. Here, the MOs shown by red characters provide a dominant contribution to the transitions. In the case of two or more dominant MOs contributing to the transitions, they are indicated by orange characters. The excitations between MOs containing Pb atomic orbitals as a main component are indicated by red asterisks. Figure 3e shows the *f* values versus the excitation energies by using a stick bar (blue: monomer, yellow: monoclinic tetramer, pink: triclinic tetramer) for the relatively intense excitations. Here, the excitation energy of all the transitions was shifted by − 0.149 eV to adjust the most intense theoretical peak position (blue) to the experimental peak position (700 nm) for the monomer (see Fig. 3d and e). Although the monoclinic and triclinic phases have hitherto been understood to absorb the visible and NIR regions, respectively^1–12^, comparison between the experimental (Fig. 3c and d) and theoretical (Fig. 3e) results indicates that (i) the shoulder band at approximately 650 nm (Fig. 3c) is attributed to not only the monoclinic but also triclinic phases (the monoclinic phase has a relatively smaller contribution rather than the triclinic phase), (ii) the intense band at approximately 750 nm (Fig. 3c) is mainly attributed not to the monoclinic phase but to the amorphous phase (isolated molecules), and (iii) the intense broad band at approximately 900 nm (Fig. 3c) is mainly attributed to the triclinic phase. The present results not only confirmed theoretically the experimental interpretation that the NIR absorption band is mainly attributed to the triclinic phase^1–12^ but also obtained new findings of (i) and (ii) and furthermore found that (iv) the absorption edge in the NIR region longer than 1000 nm is attributed only to the monoclinic phase (inset of Fig. 3e), though it has been understood that the NIR region is attributed only to the triclinic phase so far (unfortunately since the present UV–vis-NIR spectrometer can measure the absorption wavelength up to 1100 nm, the two peaks are theoretically predicted to appear at this stage). As described above, a comparison between the experimental and theoretical absorption spectra^13^ suggests that the NIR absorption band of the PbPc film after annealing is attributed to the monoclinic phase rather than the triclinic phase, although the annealed film is well known to have the triclinic phase experimentally^3,14^. This discrepancy is presumably because the optimized geometries of the monoclinic and triclinic structure models^13^ are not the same as those of the experimental bulk phases: therefore, the theoretical absorption spectra were considered to be influenced by the geometrical differences. On the other hand, the present tetramer model structures for the monoclinic and triclinic phases were constructed using the X-ray crystallographic database.

### Photoluminescence spectra

As described in the introduction, there have been many reports to discuss the performance of PbPc-based OSCs in relation to the absorption characteristics of monoclinic and triclinic PbPc crystal phases^1–11^. However, to the best of our knowledge, there have been no reports to discuss the cell performance in relation to their photoluminescence (PL) characteristics, although PL results provide important information on the exciton characteristics. This is presumably because the PL intensity of the PbPc film is too weak to be detected using conventional PL measurement systems with a monochromatized UV–vis lamp. In the present study, we succeeded in obtaining the PL spectra of the PbPc films using laser-induced static/time-resolved (TR) PL spectroscopy^21,22^.

**Figure 5:**
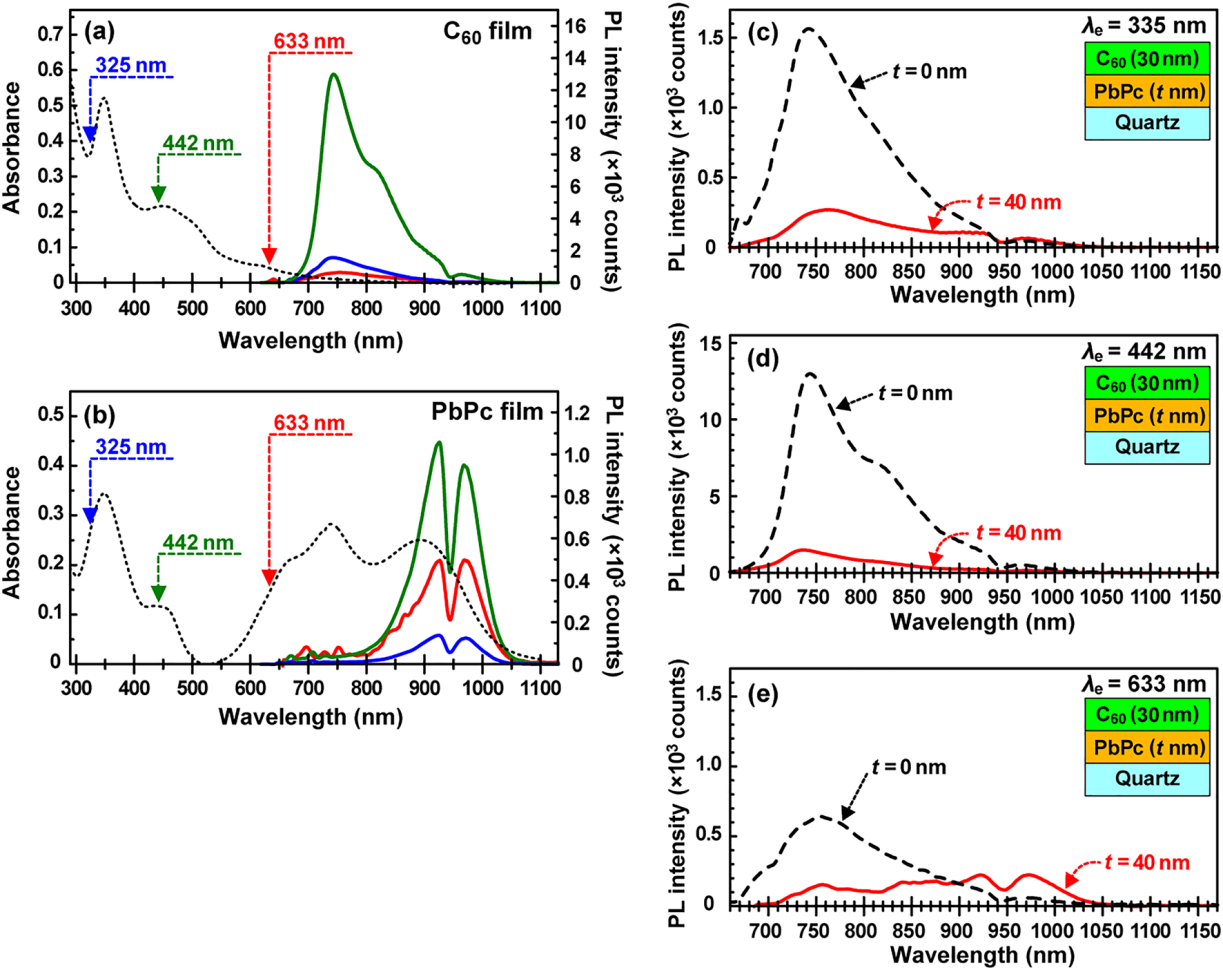
UV–vis-NIR spectra (black dashed lines) and PL spectra (blue, green, and red solid lines obtained at excitation wavelengths of 325, 425, and 633 nm, respectively) of (**a**) 30 nm-thick pristine C_60_ film and (**b**) 40 nm-thick pristine PbPc film. PL spectra of the pristine 30 nm-thick C_60_ (black dashed line) and [40 nm-thick PbPc/30 nm-thick C_60_] bilayer (red solid line) films measured at excitation wavelengths of (**c**) 325 nm, (**d**) 425 nm, and (**e**) 633 nm, respectively.

Figure 5 shows UV–vis-NIR spectra (black dashed lines) and PL spectra (blue, green, and red solid lines obtained at excitation wavelengths of 325, 425, and 633 nm, respectively) of (a) 30 nm-thick pristine C_60_ film and (b) 40 nm-thick pristine PbPc film [left], and PL spectra of pristine C_60_ (30 nm-thick) single (black dashed line) and PbPc (40 nm-thick)/C_60_ (30 nm-thick) bilayer (red solid line) films measured at excitation wavelengths of (c) 325 nm, (d) 425 nm, and (e) 633 nm, respectively [right]. The scale of the PL intensity for the PbPc film (b) is one order of magnitude smaller than that for the C_60_ film (a).

For the pristine 30 nm-thick C_60_ film with a [111]-oriented face-centered cubic structure (which is independent of the kinds of substrates such as Mica^23^, HOPG^24^, Si(111) × √3Ag^25^, gold^26^, SiO_2_^27^, etc.), lasers with wavelengths of 325, 442, and 633 nm induced the electronic transition of [HOMO-2 (*h*_*g*_)/HOMO-1 (*g*_*g*_) → LUMO (*t*_*1u*_)], [HOMO (*h*_*u*_) → LUMO + 1 (*t*_*1g*_)], and [HOMO (*h*_*u*_) → LUMO (*t*_*1u*_)], respectively^28^. Here, the symbols in parentheses denote the point-group expressions. The PL intensity depends on both the absorbance of the electronic transitions and the laser power (325 nm: 9.7 mW, 442 nm: 58.0 mW, 633: 2.7 mW) while maintaining the spectral shape. This is because the PL originates mainly from S_1_ to S_0_ for any electronic excitation^29^.

On the other hand, the PL characteristics of the 40 nm-thick PbPc film with a mixture of amorphous, monoclinic, and triclinic phases were different from those of the pristine C_60_ film. For the 40 nm thick PbPc film, it is found from XRD and UV–vis-NIR results that 20 nm lower half consists of the monoclinic and amorphous phases, and 20 nm upper half consists of the triclinic and amorphous phases. Thus, this upper half of the PbPc film contacted to the C_60_ film to form the PbPc/C_60_ interface. Accordingly, a part of the triclinic phase in the upper half is possible to contact directly to the C_60_ film at the great uneven C_60_/PbPc interface shown in Fig. S5. Considering the time scale of light passing though the bilayer film (fs), excitons are generated simultaneously in both films when compared to their PL decay at ns scale (see Fig. 7). The two distinct PL peaks originate from S_1_ to S_0_ with two levels caused by vibronic interactions (Jablonski diagram)^30–32^. As shown in Fig. 3b, the PL intensity was very weak and one order of magnitude smaller than that for the C_60_ film. In addition, although the spectral shape was invariant with the excitation wavelength, the intensity was not linearly dependent on the absorbance magnitude. As shown in Fig. S4, the PL of the isolated PbPc molecules in *N*-methylpyrrolidone (NMP) solution was observed at approximately 680 nm. Although the NMP solvent itself shows a PL of approximately 680 nm, the intensity was much weaker by 1/40 than that for the PbPc molecules, thus allowing us to be ignored.

A comparison of the PL spectra in Figs. 5b and S4a indicates that the PL of the PbPc film was not emitted from the amorphous phase (isolated molecules), thus suggesting that the PL originates from the monoclinic and triclinic phases. Since the transition probability of photoabsorption corresponds well to that of PL, the PL shown in Fig. 5b arises only from the triclinic phase (this is because the oscillator strength of the NIR peaks for the monoclinic phase is very weak and ignored when compared to that for the triclinic phase, as shown in Tables S2 and S3). Namely, even when all the phases (amorphous, monoclinic, and triclinic) were excited by irradiating the Soret band (300–500 nm) with wavelengths of 325 and 442 nm, the PL was finally emitted only via the triclinic phase. Conversely, all photogenerated excitons in the amorphous phase were transferred to the monoclinic or triclinic phase because of no corresponding PL observation, and those in the monoclinic phase were annihilated via nonradiative processes by collision between excitons^19^ (a part of excitons generated in the monoclinic and triclinic phases can be transferred from one to another). Thus, the PL efficiency becomes small, despite the laser power with wavelengths of 325 and 442 nm (9.7 and 58.0 mW, respectively) being much larger than that with 633 nm (2.7 mW). On the other hand, when the Q-band was irradiated at 633 nm, the triclinic phase dominantly contained in the region was directly excited: thus comparatively intense PL (red) was observed even when using weak laser power.

Figure 5c–e show the PL characteristics of the C_60_ (30 nm)/PbPc (40 nm) bilayer film (red solid line) with excitation wavelengths of 335, 442, and 633 nm, along with that of the pristine C_60_ film (black dashed line). For the bilayer film irradiated with 335 and 442 nm (c, d), the PL intensity attributed to both C_60_ (a) and PbPc (b) drastically decreased. This is because photogenerated excitons in both films are dissociated into holes and electrons at the C_60_/PbPc interface prior to annihilation via recombination or nonradiative processes. For the bilayer film irradiated at 633 nm (e), PL spectra corresponding to PbPc were slightly observed. This may be because a part of the photogenerated excitons in the triclinic phase were recombined to emit PL prior to reaching the interface.

### External quantum efficiency

**Figure 6:**
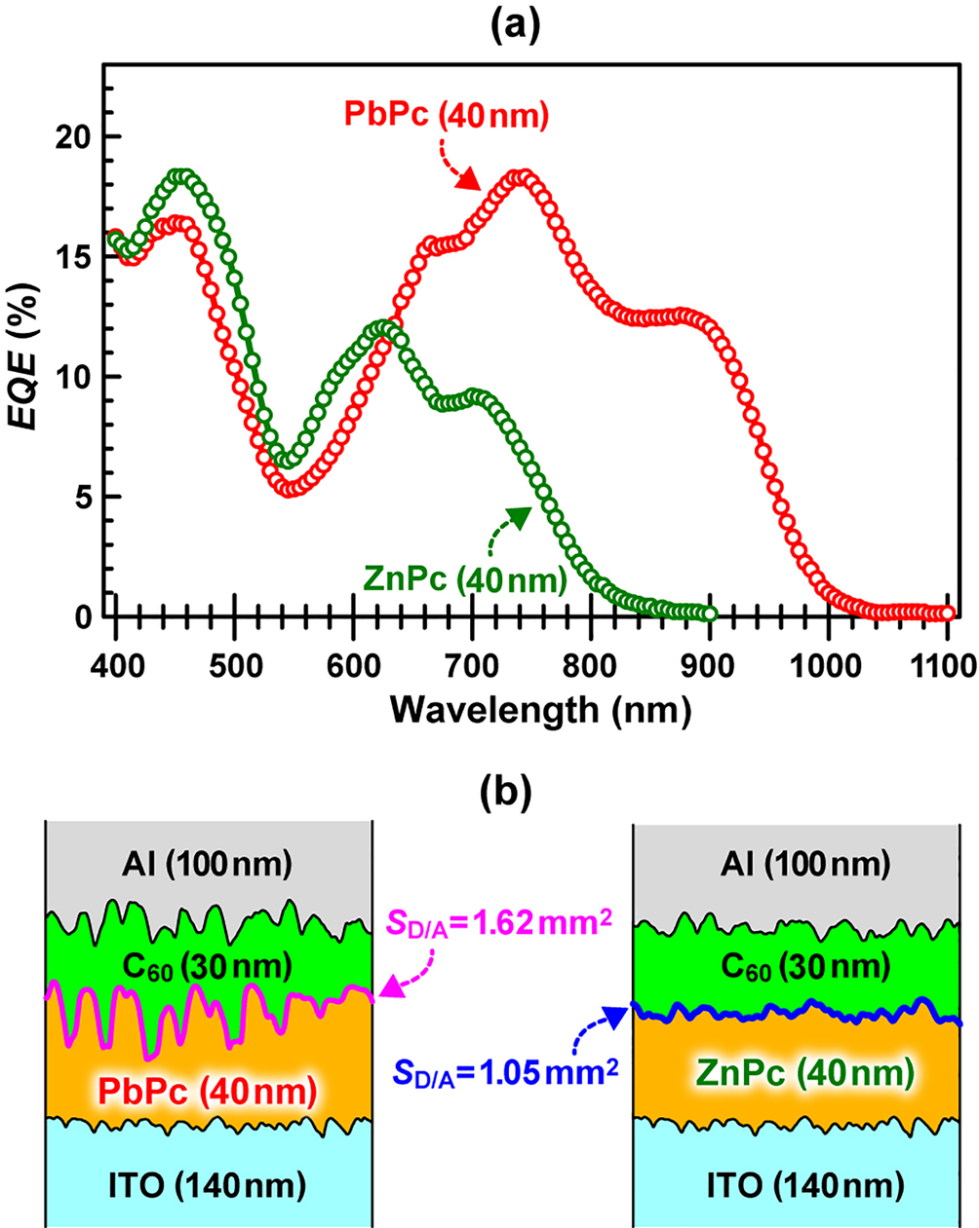
(**a**) *EQE* action spectra of [ITO/PbPc/C_60_/Al] (red) and [ITO/ZnPc/C_60_/Al] (green) OSCs and (**b**) schematic representation of both cross-sectional cell structures illustrated on the basis of AFM results (Fig. S6). The donor/acceptor effective interface area (*S*_D/A_) for PbPc/C_60_ and ZnPc/C_60_ bilayer heterojunctions with an irradiation area of 1 × 1 mm^2^ was estimated to be 1.62 and 1.05 mm^2^ from the AFM depth profiles (2 × 2 mm^2^) of PbPc and ZnPc films on ITO, respectively, by assuming that those bilayer heterojunctions are uniformly formed on ITO.

We next examined the external quantum efficiency (*EQE*) of [ITO/PbPc/C_60_/Al] and [ITO/ZnPc/C_60_/Al] OSCs and discussed the difference in their *EQE*s by comparing the structural, morphological, and optical characteristics (absorption, static and time-resolved PL) between the PbPc and ZnPc films. Figure 6 shows (a) the *EQE* action spectra of [ITO/PbPc/C_60_/Al] (red) and [ITO/ZnPc/C_60_/Al] (green) OSCs and (b) schematic representation of both cross-sectional cell structures illustrated on the basis of AFM results (Fig. S5). The donor/acceptor (D/A) effective interface area (*S*_D/A_) for the PbPc/C_60_ and ZnPc/C_60_ bilayer heterojunctions with an irradiation area of 1 × 1 mm^2^ was estimated to be 1.62 and 1.05 mm^2^ from the AFM depth profiles obtained in a measurement area of 2 × 2 mm^2^ [see the experimental section], respectively, by assuming that those bilayer heterojunctions were uniformly formed on ITO. Since a wide *S*_D/A_ generates a large number of carriers (holes and electrons) from exciton dissociation at the D/A interface such as the idea of a bulk heterojunction^33^, the wide *S*_D/A_ of the C_60_/PbPc interface is considered to compensate for the short *L*_D_ of the PbPc film. In addition, in a similar manner to [ITO/ZnPc/C_60_/Al] OSC^28^, Fig. S6 demonstrates that no reactions between PbPc and C_60_ molecules take place by confirming no changes in UV–vis-NIR spectra of the PbPc/C_60_ codeposited film before and after 24 h UV–vis photoirradiation (Fluence: 0.2 W/cm^2^), thus guaranteeing that the PbPc/C_60_ interface remained unchanged during the *EQE* measurements.

We next discuss the difference in the *EQE* between the two OSCs in the three regions of 400–600 nm, 600–800 nm, and 800–1100 nm. As shown in Fig. 6a, the *EQEs* of the ZnPc- and PbPc-based OSCs were comparable to each other in the range of 400–600 nm. Judging from the absorption spectra of the 30 nm-thick C_60_ (Fig. 5a), 40 nm-thick PbPc (Fig. 5b or Fig. S6a), and 40 nm-thick ZnPc (Fig. S6b) films, the photogenerated excitons in the C_60_ film mainly contributed to the *EQE*, thus resulting in comparable *EQE* spectra between the [ITO/PbPc/C_60_/Al] and [ITO/ZnPc/C_60_/Al] OSCs in this range.

In the range of 600–800 nm, it is found that the *EQE* of the PbPc-based OSC was significantly greater than that of the ZnPc-based OSC, although the whole absorbance of the PbPc and ZnPc films seems to be comparable to each other in this range. As shown in Fig. S7, the PbPc film exhibited a PL spectrum (red) much weaker by one order than that of the ZnPc film when both Q-bands were excited by the 633-nm laser, despite the absorbance of both films being comparable to each other at 633 nm. This suggests that a large number of photogenerated excitons in the triclinic phase were transferred to the monoclinic phase and annihilated via nonradiative processes.

This tendency became greater for the PbPc/C_60_ and ZnPc/C_60_ bilayer films, as shown in Fig. S8. These results indicate that many photogenerated excitons in the ZnPc film recombined to emit PL when compared to that in the PbPc film, which plays a crucial role in making the *EQE* of the ZnPc OSC smaller than that of the PbPc OSC. The results of Figs. S7 and S8 seem to be apparently inconsistent with the *L*_d_ (15 nm) of ZnPc being longer than that (4.6 nm) of PbPc^15,16^, because excitons with a long *L*_D_ are considered to be dissociated at the D/A interface prior to annihilation. However, the above discussion is suitable only on the assumption that the D/A interface is ideally (atomically) flat. Practically, Fig. S5 shows that the D/A bilayer interface was not a flat but an uneven structure and indicates that the PbPc/C_60_ interface exhibited uneven greater values than the ZnPc/C_60_ interface (namely, excitons with a short *L*_D_ are sufficient to reach the D/A interface with a large *S*_D/A_ prior to annihilation). Thus, the difference in the unevenness magnitude should be considered an important factor as well as *L*_d_ when the cell performances are compared.

**Figure 7:**
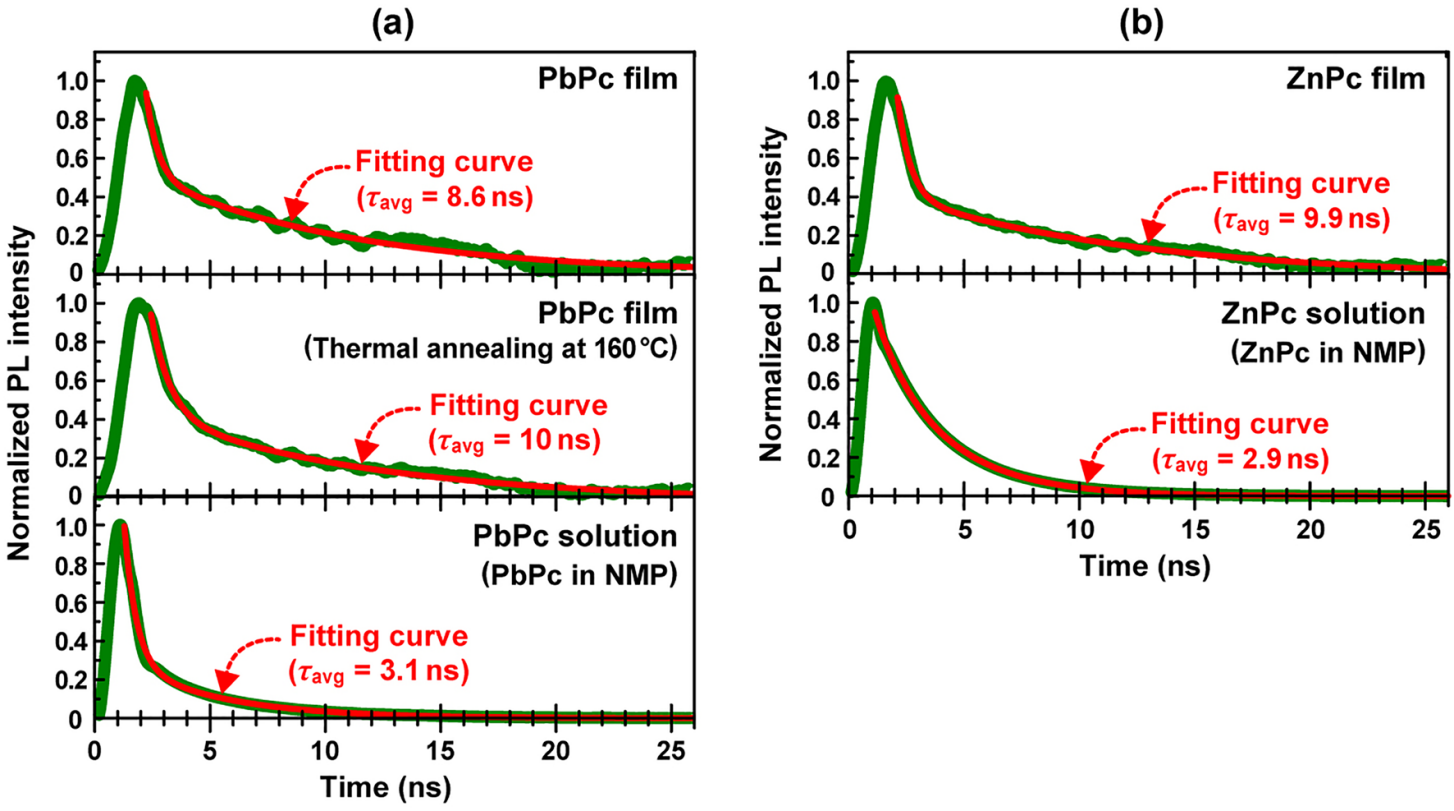
TR-PL spectra of (**a**) 40 nm-thick pristine (top) and postannealed (middle) PbPc films along with isolated PbPc molecules in NMP (bottom) and of (**b**) 40 nm-thick ZnPc film (top) and isolated ZnPc molecules in NMP (bottom). The method to estimate the average lifetime (*t*_avg_) from TR-PL spectra was described elsewhere^22^. Thermal annealing was performed at 160 °C for 1 h.

We further examined the average lifetime (*τ*_avg_) of excitons generated in the PbPc and ZnPc^21^ films with a thickness of 40 nm using TR-PL spectroscopy with an excitation wavelength of 409 nm. As shown in Fig. S7, since the PL spectra of the PbPc film overlaps with the NIR absorption band, the self-absorption should be considered for the TR-PL decay curve of the PbPc film. Judging from the absorbance of ca. 0.2 at around 950 nm, more than 80% of PL go through the film to be detected, thus suggesting that the self-absorption gives a small impact to the present results. On the other hand, since the PL spectra of the ZnPc film does not almost overlap with the absorption band, the self-absorption can be ignored. Figure 7 shows the TR-PL spectra of (a) 40 nm-thick pristine (top) and post-annealed (middle) PbPc films along with isolated PbPc molecules in NMP (bottom) and of (b) the 40 nm-thick ZnPc film (top) and isolated ZnPc molecules in NMP (bottom). Here, the black lines denote fitting curves obtained using the time-variable equation based on two kinds of relaxation lifetimes, as expressed in Eq. (1). As shown in Figs. 2 and 3, the 20 nm lower half consists of the monoclinic and amorphous phases heterogeneously, whereas the 20 nm upper half consists of the triclinic and amorphous phases heterogeneously for the 40 nm-thick PbPc film. In a similar manner, the 40 nm-thick ZnPc film formed at room temperature has the a-phase heterogeneously mixed of brickstone and herringbone structures^21^. Since the average lifetime (*τ*_avg_) is an appropriate index to compare the exciton characteristics between these highly heterogeneous structural films, we estimated *τ*_avg_ for each phase film from the fitted values of coefficients (*A*_1_ and *A*_2_) and lifetimes (*τ*_1_ and *τ*_2_) using Eq. (2).

1 $$ {\text{I (t) = A}}_{{1}} {\text{e}}^{{ - { }\frac{{\text{t}}}{{{\uptau }_{{1}} }}}} {\text{ + A}}_{{2}} {\text{e}}^{{ - { }\frac{{\text{t}}}{{{\uptau }_{{2}} }}}} $$

2 $$ {\uptau }_{{{\text{avg}}}} { = }\frac{{ {\text{A}}_{{1}} {\uptau }_{{1}} {\uptau }_{{1}} {\text{ + A}}_{{2}} {\uptau }_{{2}} {\uptau }_{{2 }} }}{{{\text{A}}_{{1}} {\uptau }_{{1}} {\text{ + A}}_{{2}} {\uptau }_{{2}} }} $$

Here the *t*_1_ denotes the fast lifetime being not affected significantly by circumstance (intermolecular interactions), whereas the *t*_2_ denotes the slow lifetime strongly affected by circumstance. Therefore, the *t*_1_ is comparable for molecule in liquid and solid phases, whereas the *t*_2_ is affected in solid phase greater than in liquid phase. Indeed, the *t*_1_ and *t*_2_ were obtained to be 0.52 and 4.16 ns for PbPc in NMP (Fig. 7a bottom), respectively, whereas 0.51 and 8.92 ns, respectively, for PbPc film (Fig. 7a top). Accordingly, the intermolecular interactions became larger to make the *t*_2_ slower in solid phase when compared to liquid phase.

Although *τ*_avg_ of both isolated molecules was almost equal to each other (PbPc: 3.1 ns, ZnPc: 2.9 ns), *τ*_avg_ (8.6 ns) of the pristine PbPc film was slightly shorter than that (9.9 ns) of the ZnPc film. This is because a part of the photogenerated excitons in the triclinic phase were transferred to the monoclinic phase and annihilated via nonradiative processes^19^ for the 40 nm-thick PbPc film, as discussed above, whereas most excitons were recombined to emit PL for the 40 nm-thick ZnPc film with a-single phase^21^. Actually, when the PbPc film was annealed at 160 °C for 1 h to change the monoclinic to triclinic phase^3,14^, *τ*_avg_ became significantly longer reaching 10.0 ns (Fig. 7a middle), which is almost equal to that of the ZnPc film.

In the NIR region of 800–1000 nm, the present study demonstrates that (I) the Q-band absorption is dominantly due to the triclinic phase (Figs. 3 and 4), and (II) this phase (and amorphous phase) contacts the C_60_ film for the 40 nm-thick PbPc film (Fig. 2). These facts allow us to consider that a large number of photogenerated excitons even with a short *L*_D_ (4.6 nm) easily diffused to (III) the uneven PbPc/C_60_ interface with a large *S*_D/A_ (Figs. 6b and S5) and dissociated to generate carriers effectively prior to annihilation via recombination or nonradiative processes. Furthermore, (IV) the delocalized electronic states of the monoclinic phase in contact with ITO are considered to enhance the mobility of holes coming from the D/A interface. Thus, the PbPc-based OSC exhibited a relatively large *EQE* in the NIR region (Fig. 6a). Accordingly, the four features (I)–(IV) of the PbPc film play a good absorber for NIR-sensitive solar cells^1–11^ and photodetectors^34^.

In summary, we have provided new findings on the assignment of the visible and NIR absorption bands of the PbPc film with a mixture of amorphous, monoclinic, and triclinic phases and revealed the dynamics of photogenerated excitons in the mixed phases by first demonstrating the static and time-resolved PL spectra of the PbPc film. By comparing the difference in the *EQE* action spectra between [ITO/PbPc/C_60_/Al] and [ITO/ZnPc/C_60_/Al] OSCs in relation to the structure, morphology, optical (absorption and PL) characteristics of the PbPc and ZnPc films in combination with first-principles calculations, we unraveled the reasons behind the PbPc film acting as a good absorber for NIR-sensitive devices.

## Methods

### Sample preparation

C_60_, PbPc, and ZnPc films were formed in an ultrahigh vacuum (UHV) chamber with a base pressure of 7.0 × 10^−7^ Pa. After thermal cleaning (553 K) of a quartz substrate in the UHV chamber, C_60_, PbPc, and ZnPc molecules were deposited on the substrate at RT by thermal evaporation of C_60_ (Matsubo Co. Ltd., 99.98% pure), PbPc (TCI, 98% pure), and ZnPc (TCI, 95% pure) powders mounted into graphite (ZnPc and PbPc) and quartz (C_60_) crucibles, respectively. The deposition rates of C_60_, PbPc, and ZnPc were 1.4, 0.42, and 0.36 nm/min at temperatures of 673, 523, and 558 K, respectively. In addition, a 70 nm-thick PbPc/C_60_ codeposited film with a ratio of PbPc:C_60_ = 1:1 was formed on the quartz substrate at RT by setting the deposition rates of PbPc and C_60_ to be 0.63 and 0.74 nm/min, respectively, and thereafter the film was irradiated with UV–vis light (Fluence: 0.2 W/cm^2^) for 24 h by using a high-pressure mercury lamp (USHIO-500D) via an infrared-light cut filter (OptoSigma HAF-50S-30H), to examine the interactions between PbPc and C_60_ at the PbPc/C_60_ bilayer interface upon photoirradiation^28^.

### X-ray diffraction measurements

The crystal structure of the PbPc films thus formed was measured by X-ray diffraction using a four-axis diffractometer with Cu-Kα1 X-rays (Bruker, D8 DISCOVER) in air. The incidence angle of X-rays to the PbPc films was 0.2° with an increment of 0.003°. Simulated XRD patterns of the PbPc monoclinic and triclinic phases were obtained using VESTA (Visualization for Electronic and Structural Analysis)^35^ with an X-ray wavelength of 1.5405 nm (Cu Kα1). The maximum peak intensity of both phases was individually normalized to unit.

### Optical measurements

The absorption spectra of the ZnPc and PbPc films were obtained at RT in air using a UV–vis-NIR spectrometer (JASCO V-730) with a resolution of 0.5 nm. For the UV–vis-NIR spectra of isolated ZnPc and PbPc molecules, ZnPc and PbPc powders were dissolved into *N*-methylpyrrolidone (NMP) solutions with a molar concentration of 8.5 × 10^−5^ M. Thereafter, they were introduced into quartz cells for each and recorded at RT by using the same spectroscopy as for the films.

Photoluminescence (PL) spectra of the PbPc and ZnPc films were recorded at RT in air using an apparatus consisting of a He–Cd laser (325 nm/9.7 mW and 442 nm/58.0 mW), a He–Ne laser (633 nm/2.7 mW), a CCD camera (Princeton Instruments, PIXIS100), and a multichannel spectrometer (Roper Scientific, SpectraPro 2300i). Figure S9 shows a schematic illustration of the experimental setup. Individual laser beams were focused by a quartz lens (focal length: 10 cm) to have a spot diameter of 0.5 mmf. The measurement duration (0.5 s) of a single PL spectrum and the number of samplings (100 counts) were kept constant for all PL measurements in a detection wavelength range of 650–1150 nm via a 630 nm long pass filter to cut the excitation laser. Time-resolved PL (TR-PL) spectra of each film were obtained at RT in air using a picosecond pulsed diode laser (Hamamatsu Photonics, PLP-10) as an excitation light source. The wavelength, pulse width, and repetition rate of the diode laser were set to 409 nm, 80 ps, and 10 MHz, respectively. A time-correlated single-photon counter (PicoQuant, PicoHarp 300, resolution: 4 ps, jitter accuracy: 12 ps) was used to measure the PL time profile (wavelength longer than 680 nm). We measured a temporal profile of the excitation pulse laser and obtained the instrument response to be ca. 160 ps, which was too short to affect the time-resolved PL spectra at ns scale in the present study.

All samples were irradiated diagonally with an incident angle of ca. 45° using the laser beams. We confirmed that very weak PL spectra were observed in the range of 650–700 nm for the bare quartz substrate irradiated diagonally with individual laser beams and they were two or three order of magnitude smaller than that for the PbPc and C_60_ film. For the PL and TR-PL spectra of isolated PbPc and ZnPc molecules, ZnPc and PbPc powders were solved in NMP with molar concentrations of 8.5 × 10^−7^ and 8.5 × 10^−5^ M, respectively. Thereafter, they were introduced into quartz cells for each and recorded at RT by using the same spectroscopy as for the films. The method to estimate the average lifetime (*t*_avg_) from TR-PL spectra was described elsewhere^36^.

### Atomic force microscope measurements

The surface morphology of ITO, ITO/PbPc, ITO/ZnPc, ITO/PbPc/C_60_, and ITO/ZnPc/C_60_ was ex situ measured at RT in a 2 × 2 mm^2^ area by atomic force microscopy (AFM: SPA400, Hitachi High-Tech Science) with a dynamic force mode, using an aluminum-coated silicon cantilever (SI-DF20, frequency: 110–130 kHz). To estimate the effective interface area (*S*_D/A_ shown in Fig. 6) of the MPc/C_60_ (M = Pb, Zn) bilayer heterojunction, we calculated the vertical and horizontal curve lengths using the line segment between two adjacent points (512 points recorded in 2 mm) for each. In addition, to estimate the error due to the linear approximation for the line segment, we smoothed the segment at five adjacent points using the Savitzky-Golay filter^37^ and then estimated those curve lengths more precisely in terms of cubic polynomial spline interpolation. Since a comparison between the two results showed the error to be slightly 1.5%, the *S*_D/A_ values for PbPc/C_60_ and ZnPc/C_60_ obtained using the linear approximation are shown in Fig. 6. Assuming that the bilayer films were formed uniformly in the active area of 1 × 1 mm^2^, the interface-area ratio of unevenness to flat obtained in the 2 × 2 mm^2^ area can be used for the present OSC with an active area of 1 × 1 mm^2^.

### OSC fabrication and external quantum efficiency measurements

Organic solar cells (OSCs) were fabricated in the same UHV chamber (base pressure: 7.0 × 10^−7^ Pa). One millimeter-wide 140 nm-thick indium tin oxide (ITO) substrates (Aldrich) were used as transparent electrodes after cleaning by air plasma exposure (1 × 10^4^ Pa, 30 mA) for 20 min. A PbPc film (20 nm thick or 40 nm thick) was formed on the ITO electrode. Thereafter, a 30 nm-thick C_60_ film was deposited on ITO/PbPc at RT. Finally, a 1 mm-wide 100 nm-thick aluminum (Al) cathode contact was formed on ITO/PbPc/C_60_ at RT by thermal evaporation of a granular Al source (Nirako, 99.999% pure) mounted into an alumina crucible. Thus, the active area of the [ITO/PbPc/C_60_/Al] solar cells was 1 × 1 mm^2^. [ITO/ZnPc/C_60_/Al] OSCs were fabricated in a similar procedure to [ITO/PbPc/C_60_/Al] OSCs.

The external quantum efficiency (*EQE*) of the solar cells thus formed was recorded at RT in air, using a lock-in amplifier (Toyo Corporation 5210, modulation range: 400–450 Hz) in a range of 400–800 nm. By adjusting the incident angle to 90° normal to the cells, the device active area of 1 × 1 mm^2^ was irradiated with parallel monochromatic light. We confirmed that the *EQE* action spectra of a silicon photodiode (Hamamatsu Photonics S1336-18BU) were the same as that of its reference data using the present measurement system.

### Theoretical calculations

The tetramer model structures for both PbPc monoclinic and triclinic crystals were constructed using the X-ray crystallographic databases of CCDC Nos. 1229291 and 1,229,292, respectively. In the case of the monoclinic model, a lack of the X-ray data was compensated by the symmetry operation. For the DFT and time-dependent (TD)-DFT calculations, the three-parameterized Becke-Lee–Yang–Parr (B3LYP) hybrid exchange–correlation functional^38^ was employed with Lanl08(d)^36^ and 6-31G* basis set^39,40^ for Pb and others (relativistic effects except spin–orbit coupling were considered). The 30 and 40 excited states (S1–S30 and S1–S40) were examined by TD-DFT calculations for the triclinic and monoclinic crystal phases, respectively. All calculations were performed in the gas phase condition by using the Gaussian 09 revision D.01 program package^41^.

## Supplementary Information

Supplementary Information.

## Data Availability

All data are available within the article and supplementary files or available from the corresponding authors on reasonable request.
