# Supplementary material for: Unraveling the reasons behind lead phthalocyanine acting as a good absorber for near-infrared sensitive devices
Source: Sci Rep. 2022 May 25;12:8810. doi: 10.1038/s41598-022-12990-z (PMC9132886; doi:10.1038/s41598-022-12990-z)
Supplement: Supplementary file 1 — Supplementary Information. [file 41598_2022_12990_MOESM1_ESM.pdf]

Supplementary materials

Unraveling the reasons behind lead phthalocyanine acting as a good absorber for near-infrared sensitive devices

Masahiro Kato, Hayato Yoshizawa, Masato Nakaya, Yasutaka Kitagawa, Koichi Okamoto, Tomoaki Yamada, Masahito Yoshino, Kentaro Tanaka, and Jun Onoe

Table S1. The wavelength [nm], oscillator strength (*f*), excitation transition from the occupied MO (S0) to the unoccupied MO (S1–S10), and relative ratio for the excitations of the monomer in solvent (top) and in gas phase (bottom). The MOs shown by red characters provide a dominant contribution to the transitions. In case of two or more dominant MOs contributing to the transitions, they are indicated by orange characters. The solvent effects were approximated in terms of methanol with the polarizable continuum model (PCM) by using the integral equation formalism variant (IEFPCM).

Monomer (solvent = methanol)

S_n	nm	*f*	From	To	ratio
1	648.19	0.4506	HOMO-5	LUMO+1	2%
			HOMO-1	LUMO+1	3%
			HOMO	LUMO	95%
2	648.19	0.4506	HOMO-5	LUMO	2%
			HOMO-1	LUMO	3%
			HOMO	LUMO+1	95%
3	436.21	0.0697	HOMO-5	LUMO	3%
			HOMO-1	LUMO	91%
			HOMO	LUMO+1	3%
4	436.21	0.0697	HOMO-5	LUMO+1	3%
			HOMO-1	LUMO+1	91%
			HOMO	LUMO	3%
5	380.14	0.0030	HOMO-2	LUMO	94%
6	380.14	0.0030	HOMO-2	LUMO+1	94%
9	362.14	0.0133	HOMO-9	LUMO	66%
			HOMO-8	LUMO	32%
10	362.14	0.0133	HOMO-9	LUMO+1	66%
			HOMO-8	LUMO+1	32%

Table S1 continued to next page

Monomer (gas)

S_n	nm	*f*	From	To	ratio
1	629.61	0.3299	HOMO-3	LUMO+1	3%
			HOMO-1	LUMO+1	4%
			HOMO	LUMO	93%
2	629.61	0.3299	HOMO-3	LUMO	3%
			HOMO-1	LUMO	4%
			HOMO	LUMO+1	93%
3	432.16	0.0525	HOMO-3	LUMO	4%
			HOMO-1	LUMO	90%
			HOMO	LUMO+1	3%
4	432.16	0.0525	HOMO-3	LUMO+1	4%
			HOMO-1	LUMO+1	90%
			HOMO	LUMO	3%
5	377.85	0.0016	HOMO-2	LUMO	94%
6	377.85	0.0016	HOMO-2	LUMO+1	94%
9	368.69	0.0062	HOMO-9	LUMO	49%
			HOMO-6	LUMO	49%
10	368.69	0.0062	HOMO-9	LUMO+1	49%
			HOMO-6	LUMO+1	49%

Table S2. The wavelength, oscillator strength, excitation transition from the occupied MO (S0) to the unoccupied MO (S1–S40), and relative ratio for the excitations of the monoclinic tetramer. The MOs shown by red characters provide a dominant contribution to the transition. In case of two or more dominant MOs contributing to the transition, they are indicated by orange characters. The excitations between Mos containing the Pb atomic orbitals as a main component are indicated by red asterisks.

S_n	nm	*f*	From	To	ratio
3	1074.7	0.0008	HOMO-1	LUMO	2%
			HOMO	LUMO+2	95%
4	1074.6	0.0008	HOMO-1	LUMO+1	2%
			HOMO	LUMO+3	95%
5	997.53	0.0007	HOMO-1	LUMO	91%
6	997.37	0.0007	HOMO-1	LUMO+1	91%
7	924.63	0.0001	HOMO-3	LUMO+2	5%
			HOMO-2	LUMO	4%
			HOMO-1	LUMO+2	16%
			HOMO	LUMO+4	72%
8	924.5	0.0001	HOMO-3	LUMO+3	5%
			HOMO-2	LUMO+1	4%
			HOMO-1	LUMO+3	16%
			HOMO	LUMO+5	72%
9	879.7	0.0001	HOMO-2	LUMO	22%
			HOMO-1	LUMO+2	59%
			HOMO-1	LUMO+6	5%
			HOMO	LUMO+4	8%
10	879.58	0.0001	HOMO-2	LUMO+1	22%
			HOMO-1	LUMO+3	59%
			HOMO-1	LUMO+7	5%
			HOMO	LUMO+5	8%
11	835.58	0.0002	HOMO-3	LUMO	29%
			HOMO-2	LUMO	10%
			HOMO-2	LUMO+2	7%
			HOMO-1	LUMO+2	7%
			HOMO-1	LUMO+4	2%
			HOMO	LUMO+4	3%
			HOMO	LUMO+6	38%
12	835.47	0.0002	HOMO-3	LUMO+1	29%
			HOMO-2	LUMO+1	10%
			HOMO-2	LUMO+3	7%
			HOMO-1	LUMO+3	7%
			HOMO-1	LUMO+5	2%
			HOMO	LUMO+5	3%
			HOMO	LUMO+7	38%
13	826.14	0.0002	HOMO-3	LUMO	11%
			HOMO-2	LUMO	49%
			HOMO-2	LUMO+4	4%
			HOMO-1	LUMO+2	10%
			HOMO-1	LUMO+4	5%
			HOMO-1	LUMO+6	3%
			HOMO	LUMO+4	9%
			HOMO	LUMO+6	6%
14	826.03	0.0002	HOMO-3	LUMO+1	11%
			HOMO-2	LUMO+1	49%
			HOMO-2	LUMO+5	4%
			HOMO-1	LUMO+3	10%
			HOMO-1	LUMO+5	5%
			HOMO-1	LUMO+7	3%
			HOMO	LUMO+5	9%
			HOMO	LUMO+7	6%
15	764.96	0.0004	HOMO-3	LUMO	2%
			HOMO-2	LUMO+2	3%
			HOMO-1	LUMO+4	62%
			HOMO	LUMO+6	22%
16	764.87	0.0004	HOMO-3	LUMO+1	2%
			HOMO-2	LUMO+3	3%
			HOMO-1	LUMO+5	62%
			HOMO	LUMO+7	22%
17	745.19	0.0003	HOMO-4	LUMO	5%
			HOMO-3	LUMO	27%
			HOMO-2	LUMO	3%
			HOMO-2	LUMO+2	49%
			HOMO-1	LUMO+6	3%
			HOMO	LUMO+6	4%
18	745.11	0.0003	HOMO-4	LUMO+1	5%
			HOMO-3	LUMO+1	27%
			HOMO-2	LUMO+1	3%
			HOMO-2	LUMO+3	49%
			HOMO-1	LUMO+7	3%
			HOMO	LUMO+7	4%
★19	721.05	0.0048	HOMO-6	LUMO	2%
			HOMO-5	LUMO	6%
			HOMO-4	LUMO	63%
			HOMO-4	LUMO+2	5%
			HOMO-4	LUMO+4	5%
			HOMO-3	LUMO+2	6%
			HOMO-2	LUMO+2	3%
★20	720.97	0.0048	HOMO-6	LUMO+1	2%
			HOMO-5	LUMO+1	6%
			HOMO-4	LUMO+1	63%
			HOMO-4	LUMO+3	5%
			HOMO-4	LUMO+5	5%
			HOMO-3	LUMO+3	6%
			HOMO-2	LUMO+3	3%
★21	715.93	0.033	HOMO-6	LUMO	37%
			HOMO-6	LUMO+2	5%
			HOMO-5	LUMO	27%
			HOMO-5	LUMO+2	3%
			HOMO-4	LUMO	5%
			HOMO-2	LUMO+2	6%
			HOMO-1	LUMO+4	3%
★22	715.86	0.033	HOMO-6	LUMO+1	37%
			HOMO-6	LUMO+3	5%
			HOMO-5	LUMO+1	27%
			HOMO-5	LUMO+3	3%
			HOMO-4	LUMO+1	5%
			HOMO-2	LUMO+3	6%
			HOMO-1	LUMO+5	3%
★23	704.32	0.0148	HOMO-6	LUMO	15%
			HOMO-6	LUMO+2	7%
			HOMO-5	LUMO	12%
			HOMO-5	LUMO+2	20%
			HOMO-5	LUMO+4	2%
			HOMO-4	LUMO	3%
			HOMO-3	LUMO+2	20%
			HOMO-1	LUMO+6	5%
			HOMO-1	LUMO+7	3%
			HOMO	LUMO+6	2%
★24	704.25	0.0149	HOMO-6	LUMO+1	15%
			HOMO-6	LUMO+3	7%
			HOMO-5	LUMO+1	12%
			HOMO-5	LUMO+3	20%
			HOMO-5	LUMO+5	2%
			HOMO-4	LUMO+1	3%
			HOMO-3	LUMO+3	20%
			HOMO-1	LUMO+6	3%
			HOMO-1	LUMO+7	5%
			HOMO	LUMO+7	2%
25	699.64	0.0132	HOMO-6	LUMO	3%
			HOMO-6	LUMO+2	4%
			HOMO-5	LUMO	12%
			HOMO-5	LUMO+2	9%
			HOMO-3	LUMO+2	30%
			HOMO-2	LUMO+2	4%
			HOMO-1	LUMO+6	29%

Table S2 continued to next page

26	699.57	0.0133	HOMO-6	LUMO+1	3%
			HOMO-6	LUMO+3	4%
			HOMO-5	LUMO+1	12%
			HOMO-5	LUMO+3	9%
			HOMO-3	LUMO+3	30%
			HOMO-2	LUMO+3	4%
			HOMO-1	LUMO+7	29%
27	662.65	0.0001	HOMO-4	LUMO+3	2%
			HOMO-3	LUMO+2	10%
			HOMO-2	LUMO+4	59%
			HOMO-1	LUMO+6	17%
28	662.59	0.0001	HOMO-4	LUMO+2	2%
			HOMO-3	LUMO+3	10%
			HOMO-2	LUMO+5	58%
			HOMO-1	LUMO+7	17%
29	649.86	0.0568	HOMO-7	LUMO+1	4%
			HOMO-7	LUMO+3	2%
			HOMO-5	LUMO	4%
			HOMO-5	LUMO+1	2%
			HOMO-4	LUMO+2	2%
			HOMO-3	LUMO	7%
			HOMO-3	LUMO+2	10%
			HOMO-3	LUMO+4	8%
			HOMO-2	LUMO	4%
			HOMO-2	LUMO+4	14%
			HOMO-1	LUMO+6	18%
30	649.83	0.0568	HOMO-7	LUMO	4%
			HOMO-7	LUMO+2	2%
			HOMO-5	LUMO	2%
			HOMO-5	LUMO+1	4%
			HOMO-4	LUMO+3	2%
			HOMO-3	LUMO+1	7%
			HOMO-3	LUMO+3	10%
			HOMO-3	LUMO+5	8%
			HOMO-2	LUMO+1	4%
			HOMO-2	LUMO+5	14%
			HOMO-1	LUMO+7	18%
31	633.21	0.0588	HOMO-6	LUMO	4%
			HOMO-6	LUMO+4	2%
			HOMO-5	LUMO	2%
			HOMO-4	LUMO	2%
			HOMO-4	LUMO+2	27%
			HOMO-4	LUMO+3	3%
			HOMO-4	LUMO+6	4%
			HOMO-3	LUMO+4	23%
			HOMO-3	LUMO+5	6%
			HOMO-2	LUMO+4	5%
			HOMO-1	LUMO+4	3%
31	633.21	0.0588	HOMO-6	LUMO	4%
			HOMO-6	LUMO+4	2%
			HOMO-5	LUMO	2%
			HOMO-4	LUMO	2%
			HOMO-4	LUMO+2	27%
			HOMO-4	LUMO+3	3%
			HOMO-4	LUMO+6	4%
			HOMO-3	LUMO+4	23%
			HOMO-3	LUMO+5	6%
			HOMO-2	LUMO+4	5%
			HOMO-1	LUMO+4	3%
33	625.25	0.0009	HOMO-6	LUMO+4	5%
			HOMO-5	LUMO	6%
			HOMO-4	LUMO	6%
			HOMO-4	LUMO+2	30%
			HOMO-4	LUMO+3	2%
			HOMO-4	LUMO+6	3%
			HOMO-3	LUMO+2	3%
			HOMO-3	LUMO+4	12%
			HOMO-3	LUMO+5	4%
			HOMO-2	LUMO+6	7%
			HOMO-2	LUMO+7	4%
			HOMO-1	LUMO+6	4%
34	625.21	0.0009	HOMO-6	LUMO+5	4%
			HOMO-5	LUMO+1	6%
			HOMO-4	LUMO+1	6%
			HOMO-4	LUMO+2	2%
			HOMO-4	LUMO+3	30%
			HOMO-4	LUMO+7	3%
			HOMO-3	LUMO+3	3%
			HOMO-3	LUMO+4	4%
			HOMO-3	LUMO+5	12%
			HOMO-2	LUMO+6	4%
			HOMO-2	LUMO+7	7%
			HOMO-1	LUMO+7	4%
35	619.08	0.2742	HOMO-6	LUMO	3%
			HOMO-6	LUMO+1	2%
			HOMO-6	LUMO+2	4%
			HOMO-6	LUMO+4	2%
			HOMO-5	LUMO+2	6%
			HOMO-5	LUMO+4	4%
			HOMO-4	LUMO+2	4%
			HOMO-3	LUMO	7%
			HOMO-3	LUMO+4	11%
			HOMO-2	LUMO+2	11%
			HOMO-2	LUMO+6	3%
			HOMO-1	LUMO+4	5%
			HOMO	LUMO+6	12%
36	619.05	0.2741	HOMO-6	LUMO	2%
			HOMO-6	LUMO+1	3%
			HOMO-6	LUMO+3	4%
			HOMO-6	LUMO+5	2%
			HOMO-5	LUMO+3	6%
			HOMO-5	LUMO+5	4%
			HOMO-4	LUMO+3	4%
			HOMO-3	LUMO+1	7%
			HOMO-3	LUMO+5	11%
			HOMO-2	LUMO+3	11%
			HOMO-2	LUMO+7	3%
			HOMO-1	LUMO+5	5%
			HOMO	LUMO+7	12%
37	608.82	0.031	HOMO-6	LUMO+4	3%
			HOMO-5	LUMO+2	4%
			HOMO-5	LUMO+3	3%
			HOMO-4	LUMO+2	3%
			HOMO-4	LUMO+3	3%
			HOMO-3	LUMO+4	19%
			HOMO-2	LUMO+6	49%
			HOMO-1	LUMO+4	4%
38	608.78	0.0309	HOMO-6	LUMO+5	3%
			HOMO-5	LUMO+2	3%
			HOMO-5	LUMO+3	4%
			HOMO-4	LUMO+2	2%
			HOMO-4	LUMO+3	3%
			HOMO-3	LUMO+5	19%
			HOMO-2	LUMO+7	49%
			HOMO-1	LUMO+5	4%
★39	604.44	0.0922	HOMO-6	LUMO	3%
			HOMO-6	LUMO+2	33%
			HOMO-6	LUMO+4	3%
			HOMO-5	LUMO+2	5%
			HOMO-5	LUMO+4	17%
			HOMO-3	LUMO	2%
			HOMO-3	LUMO+4	3%
			HOMO-2	LUMO+6	3%
			HOMO-1	LUMO+4	2%
			HOMO	LUMO+6	4%
★40	604.4	0.0917	HOMO-6	LUMO+1	3%
			HOMO-6	LUMO+3	33%
			HOMO-6	LUMO+5	3%
			HOMO-5	LUMO+3	5%
			HOMO-5	LUMO+5	17%
			HOMO-3	LUMO+1	2%
			HOMO-3	LUMO+5	3%
			HOMO-2	LUMO+7	3%
			HOMO-1	LUMO+5	2%
			HOMO	LUMO+7	4%

Table S3. The wavelength, oscillator strength, excitation transition from the occupied MO (S0) to the unoccupied MO (S1–S30), and relative ratio for S1–S30 excitations of the triclinic tetramer. The MOs shown by red characters provide a dominant contribution to the transitions. The excitations between Mos containing the Pb atomic orbitals as a main component are indicated by red asterisks.

S_n	nm	f	From	To	ratio
1	841.67	0.2749	HOMO-3	LUMO+3	3%
			HOMO-1	LUMO+3	5%
			HOMO	LUMO	86%
			HOMO	LUMO+2	3%
3	824.41	0.0922	HOMO-3	LUMO+1	4%
			HOMO-1	LUMO+1	31%
			HOMO	LUMO+2	58%
6	790.18	0.1941	HOMO-2	LUMO	5%
			HOMO-2	LUMO+2	4%
			HOMO-1	LUMO+1	30%
			HOMO-1	LUMO+3	46%
			HOMO	LUMO+2	10%
			HOMO	LUMO+6	2%
8	761.11	0.0238	HOMO-1	LUMO+1	25%
			HOMO-1	LUMO+3	32%
			HOMO	LUMO	6%
			HOMO	LUMO+2	19%
			HOMO	LUMO+4	11%
9	740.78	0.1978	HOMO-3	LUMO+3	3%
			HOMO-2	LUMO+2	5%
			HOMO-1	LUMO+1	5%
			HOMO-1	LUMO+5	19%
			HOMO	LUMO+4	57%
11	728.26	0.0151	HOMO-3	LUMO+1	3%
			HOMO-2	LUMO	5%
			HOMO-1	LUMO+1	3%
			HOMO-1	LUMO+5	12%
			HOMO	LUMO+4	10%
			HOMO	LUMO+6	60%
14	697.91	0.0606	HOMO-2	LUMO	4%
			HOMO-2	LUMO+2	2%
			HOMO-2	LUMO+4	5%
			HOMO-2	LUMO+6	3%
			HOMO-1	LUMO+5	16%
			HOMO-1	LUMO+7	59%
			HOMO	LUMO+4	3%
			HOMO	LUMO+6	2%
15	676.16	0.0022	HOMO-2	LUMO	7%
			HOMO-1	LUMO+3	3%
			HOMO-1	LUMO+5	34%
			HOMO-1	LUMO+7	16%
			HOMO	LUMO+4	11%
			HOMO	LUMO+6	25%
18	655.57	0.0101	HOMO-3	LUMO+1	5%
			HOMO-3	LUMO+3	7%
			HOMO-2	LUMO	57%
			HOMO-2	LUMO+2	9%
			HOMO-1	LUMO+3	6%
			HOMO-1	LUMO+5	7%
			HOMO-1	LUMO+7	6%
20	638.6	0.0842	HOMO-3	LUMO+1	60%
			HOMO-3	LUMO+3	4%
			HOMO-2	LUMO	6%
			HOMO-2	LUMO+2	7%
			HOMO-1	LUMO+1	2%
			HOMO-1	LUMO+7	3%
			HOMO	LUMO+2	4%
			HOMO	LUMO+6	3%
22	621.97	0.1379	HOMO-3	LUMO+1	16%
			HOMO-2	LUMO	8%
			HOMO-2	LUMO+2	63%
			HOMO-2	LUMO+4	3%
			HOMO-1	LUMO+7	3%
24	612.8	0.4304	HOMO-3	LUMO+3	66%
			HOMO-2	LUMO+2	3%
			HOMO-2	LUMO+4	8%
			HOMO-2	LUMO+6	5%
			HOMO	LUMO	2%
26	607.18	0.0295	HOMO-3	LUMO+1	5%
			HOMO-3	LUMO+3	6%
			HOMO-3	LUMO+5	24%
			HOMO-2	LUMO+4	52%
			HOMO-1	LUMO+7	4%
28	586.38	0.3172	HOMO-3	LUMO+3	5%
			HOMO-3	LUMO+5	26%
			HOMO-2	LUMO+4	7%
			HOMO-2	LUMO+6	47%
			HOMO-1	LUMO+5	3%
			HOMO-1	LUMO+7	4%
30	570.76	0.2022	HOMO-3	LUMO+5	18%
			HOMO-3	LUMO+7	40%
			HOMO-2	LUMO+4	14%
			HOMO-2	LUMO+6	19%
			HOMO	LUMO+4	3%

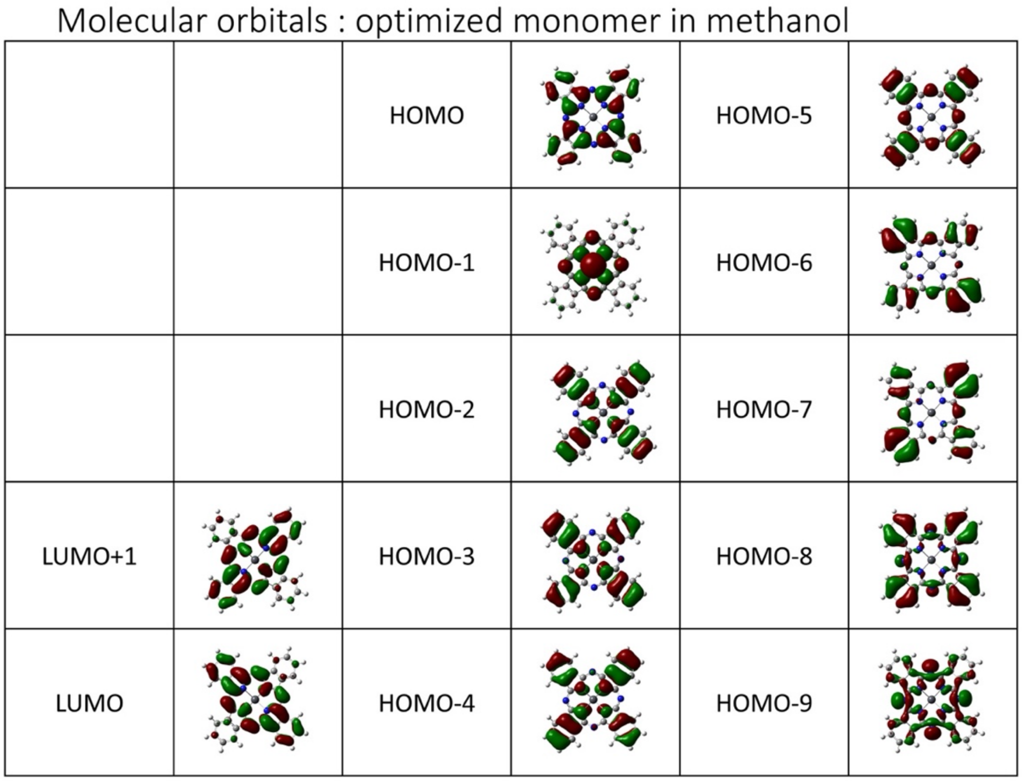

Fig. S1. Schematic representation of the frontier molecular orbital wavefunctions for the optimized PbPc monomer in methanol.

Molecular orbitals : Monoclinic 4mer

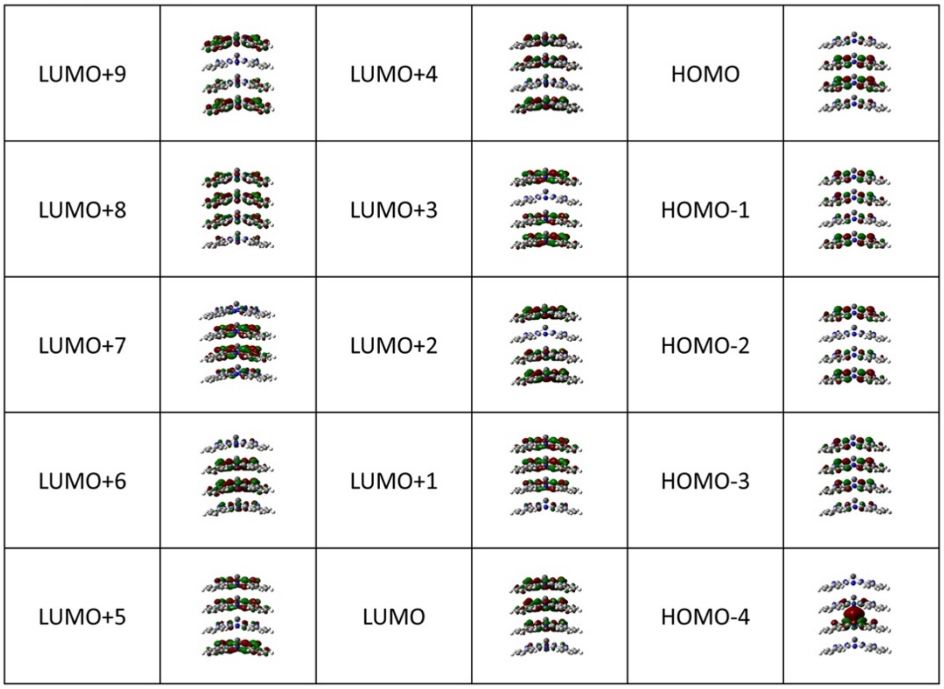

Molecular orbitals : monoclinic 4mer

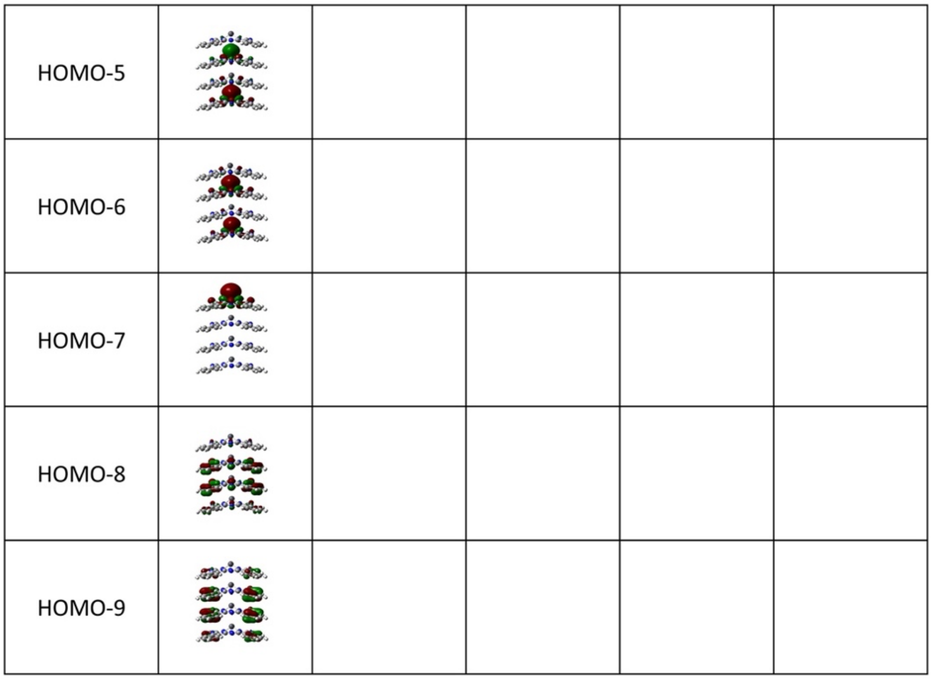

Fig. S2. Schematic representation of the frontier molecular orbital wavefunctions for the PbPc monoclinic tetramer.

Molecular orbitals : Triclinic 4mer

LUMO+9	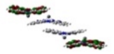	LUMO+4	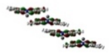	HOMO	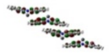
LUMO+8	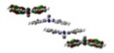	LUMO+3	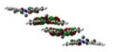	HOMO-1	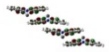
LUMO+7	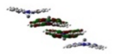	LUMO+2	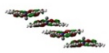	HOMO-2	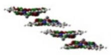
LUMO+6	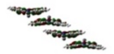	LUMO+1	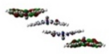	HOMO-3	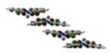
LUMO+5	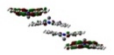	LUMO	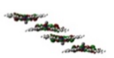	HOMO-4	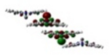

Molecular orbitals : Triclinic 4mer

HOMO-5	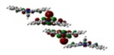				
HOMO-6	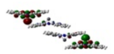				
HOMO-7	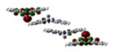				
HOMO-8	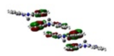				
HOMO-9	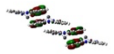				

Fig. S3. Schematic representation of the frontier molecular orbital wavefunctions for the PbPc triclinic tetramer.

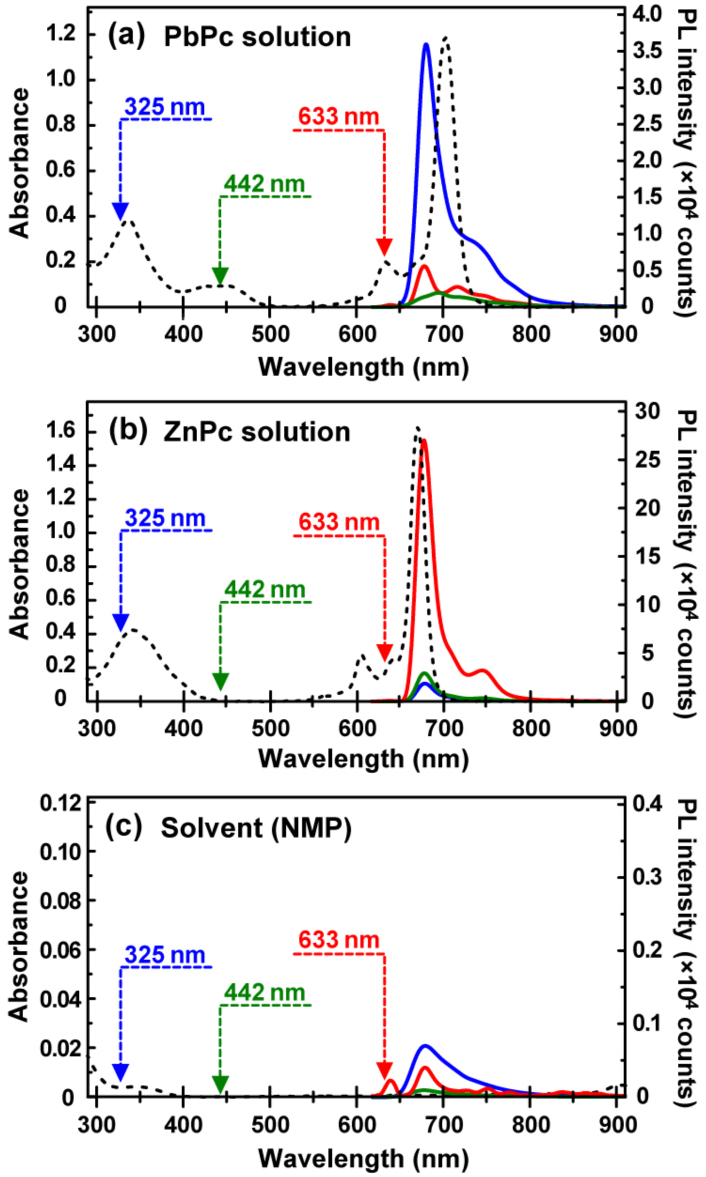

Fig. S4. Absorption (black dashed line) and PL spectra (blue, green, and red solid lines) of (a) PbPc in NMP, (b) ZnPc in NMP, and (c) NMP itself. The blue, green, and red PL spectra were recorded with an excitation wavelength of 325, 425, and 633 nm, respectively.

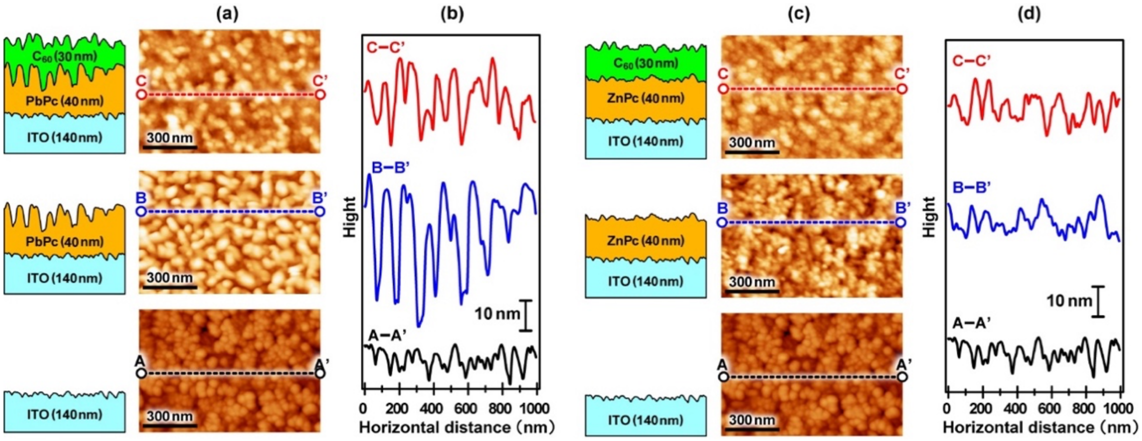

Fig. S5. AFM image and depth profile of 140 nm-thick ITO, 40 nm-thick PbPc film on ITO, and 30 nm-thick C_{60} film on PbPc/ITO (a, b), and those of 140 nm-thick ITO, 40 nm-thick ZnPc film on ITO, and 30 nm-thick C_{60} film on ZnPc/ITO (c, d). Here, the dashed line in each AFM image corresponds to the solid curve in each depth profile for each color.

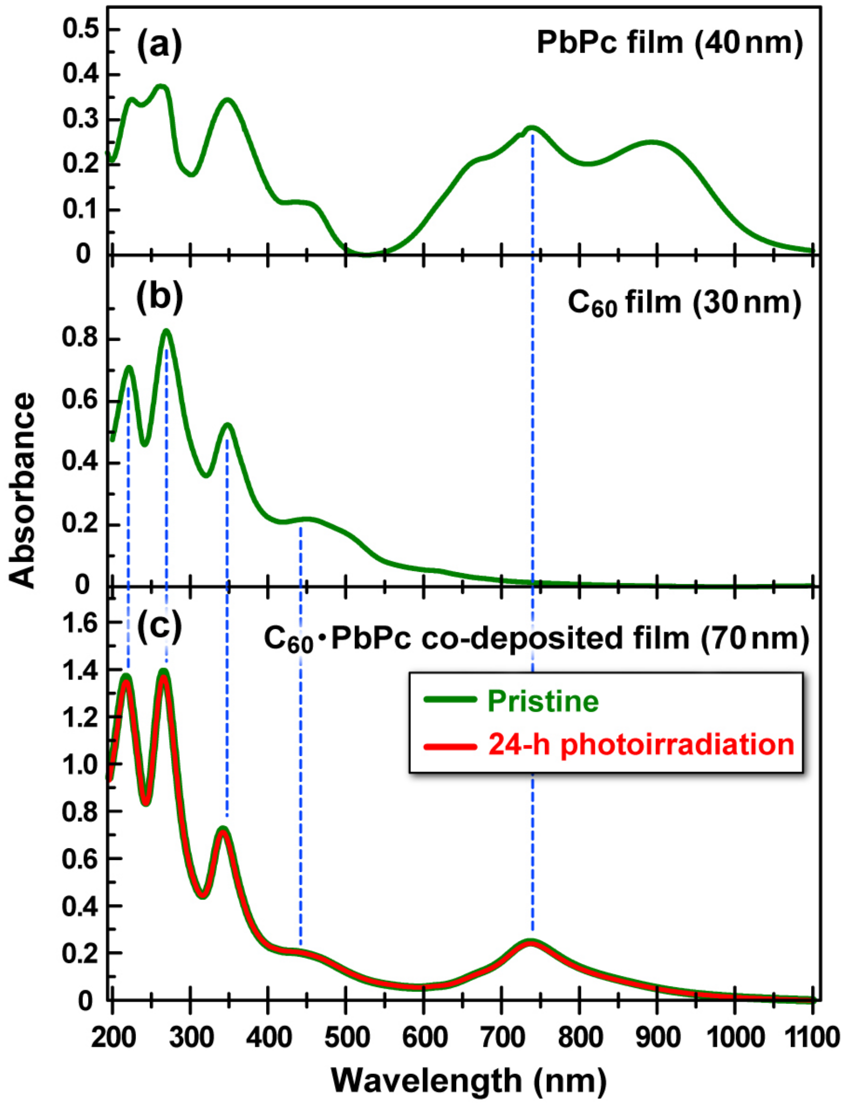

Fig. S6. UV-vis-NIR spectra of (a) 40 nm-thick PbPc pristine film, (b) 30 nm-thick C_{60} pristine film, and (c) 70 nm-thick C_{60}/PbPc co-deposited film before (green) and after (red) 24-h UV-vis photoirradiation (Fluence: 0.2 Wcm^{-2}).

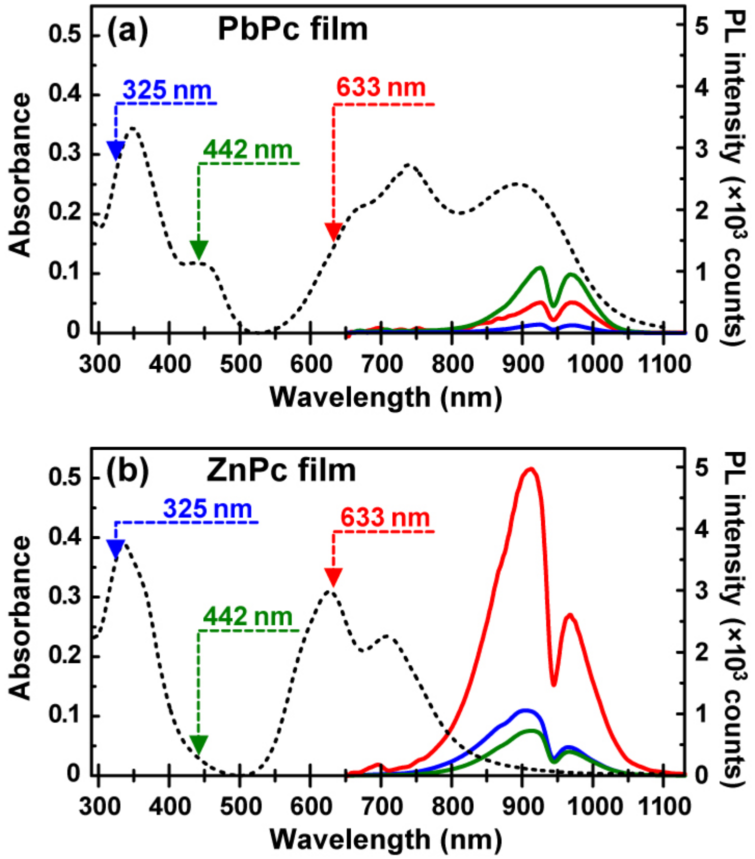

Fig. S7. Absorption (black dashed line) and PL (blue, green, and red solid lines) spectra of 40 nm-thick PbPc (a) and ZnPc (b) films. The blue, green, and red PL spectra were obtained using an excitation wavelength of 325, 425, and 633 nm, respectively.

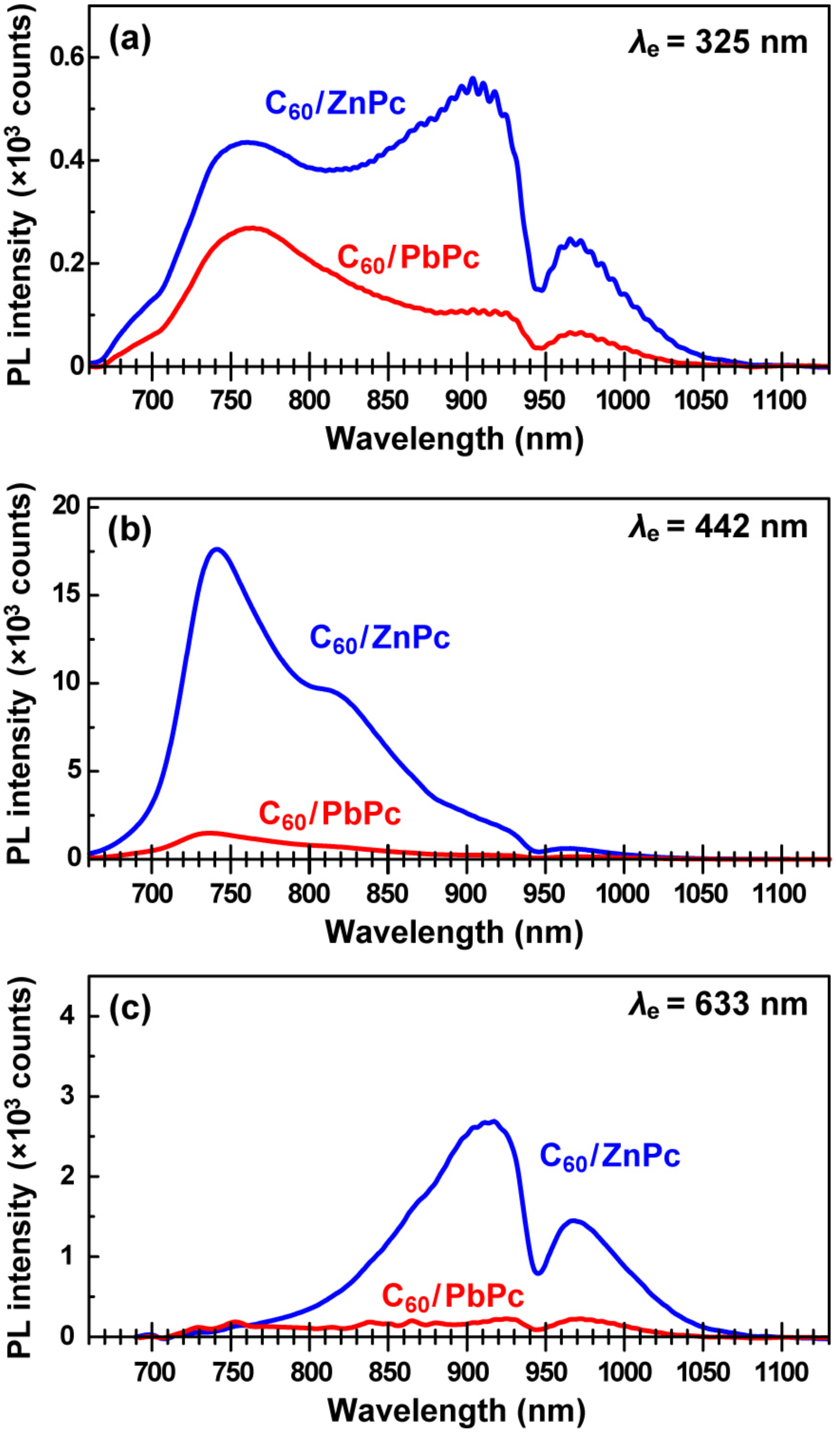

Fig. S8. PL spectra of 30 nm-C_{60}/40-nm ZnPc (blue) and 30 nm-C_{60}/40 nm-PbPc (red) bilayer films obtained by laser with a wavelength of 325 nm (a), 425 nm (b), and 633 nm (c).

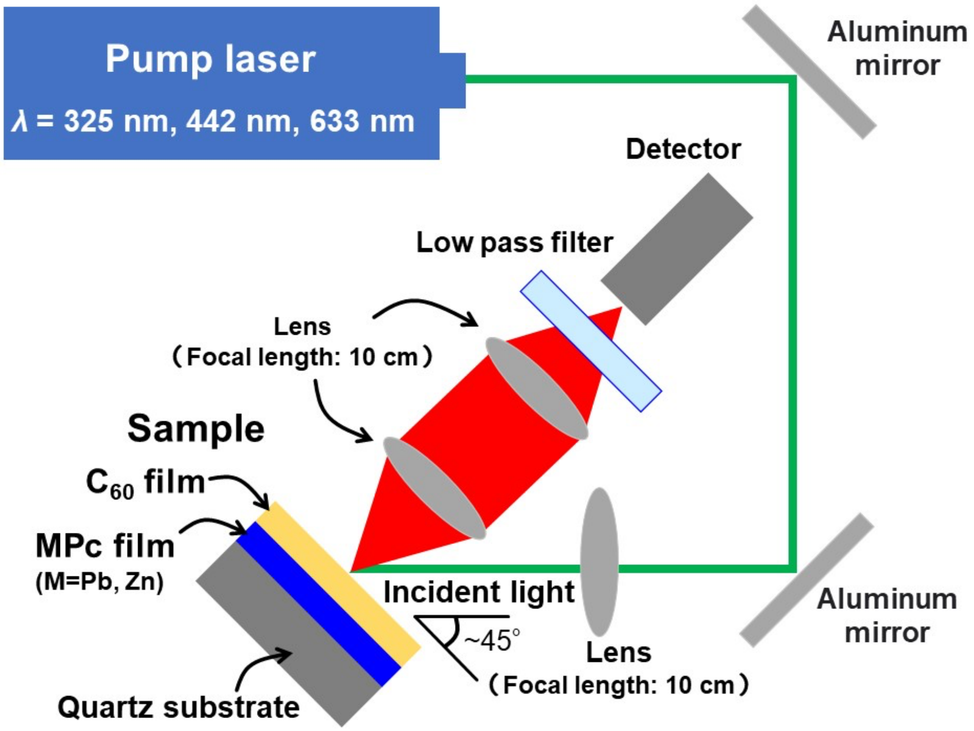

Fig. S9. Schematic illustration of photoluminescence (PL) measurement system. Individual laser beams were focused by a quartz lens (focal length: 10 cm) to have a spot diameter of 0.5 mmϕ. The measurement duration (0.5 s) of a single PL spectrum and the number of samplings (100 counts) were kept constant for all PL measurements in a detection wavelength range of 650–1150 nm *via* a 630 nm long pass filter to cut the excitation laser. For Time-resolved PL (TR-PL) measurements, we used a picosecond pulsed diode laser (Hamamatsu Photonics, PLP-10) as an excitation light source. The wavelength, pulse width, and repetition rate of the diode laser were set to 409 nm, 80 ps, and 10 MHz, respectively. A time-correlated single-photon counter (PicoQuant, PicoHarp 300, resolution: 4 ps, jitter accuracy: 12 ps) was used to measure the PL time profile (wavelength longer than 680 nm).
